# NIR nanoprobe-facilitated cross-referencing manifestation of local disease biology for dynamic therapeutic response assessment†

**DOI:** 10.1039/c9sc04909f

**Published:** 2019-11-27

**Authors:** Zhimin Wang, Xiangzhao Ai, Zhijun Zhang, Yong Wang, Xiangyang Wu, Richard Haindl, Edwin K. L. Yeow, Wolfgang Drexler, Mingyuan Gao, Bengang Xing

**Affiliations:** Division of Chemistry and Biological Chemistry, School of Physical & Mathematical Sciences, Nanyang Technological University Singapore 637371 Singapore bengang@ntu.edu.sg; Center for Molecular Imaging and Nuclear Medicine, School for Radiological and Interdisciplinary Sciences (RAD-X), Soochow University Suzhou 215123 China; Center for Medical Physics and Biomedical Engineering, Medical University of Vienna 1090 Vienna Austria

## Abstract

Pharmacological interventions for effective treatment require opportune, dynamic and accurate manifestation of pathological status. Traditional clinical techniques relying on biopsy-based histological examinations and blood tests are dramatically restricted due to their invasiveness, unsatisfactory precision, non-real-time reporting and risk of complications. Although current strategies through molecular imaging enable non-invasive and spatiotemporal mapping of pathological changes in intact organisms, environment-activatable, sensitive and quantitative sensing platforms, especially for dynamic feedback of the therapeutic response, are still urgently desired in practice. Herein, we innovatively integrate deep-tissue penetrable multispectral optoacoustic tomography (MSOT) and near-infrared (NIR) optical imaging based technology by tailoring a free radical-responsive chromophore with photon-upconverting nanocrystals. During the therapeutic process, the specific reactions between the drug-stimulated reactive oxygen species (ROS) and radical-sensitive probes result in an absorption shift, which can be captured by MSOT. Meanwhile, the radical-triggered reaction also induces multispectral upconversion luminescence (UCL) responses that exhibit the opposite trend in comparison to MSOT. Such reversed-ratiometric dual-modal imaging outcomes provide an ideal cross-referencing system that guarantees the maximum sensing specificity and sensitivity, thus enabling precise disease biology evaluation and treatment assessments *in vivo*.

## Abstract 2

NIR photoacoustic and upconversion luminescent nanoprobe-facilitated cross-referencing manifestation of oxidative stress-induced liver pathophysiology for dynamic therapeutic response assessment.

## Graphical abstract

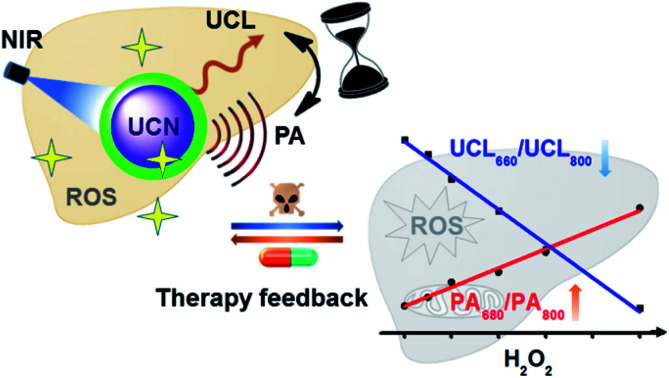

## Introduction

The growing prevalence of various diseases such as tumors, infections, cardiovascular diseases and other metabolic illnesses represents one of the leading global burdens owing to their substantial impacts on public health.^1–4^ Due to the occult symptoms and pathological complexity, effective treatment outcomes require not only advances in pharmaceutical discovery, but also innovative diagnostics for dynamic and accurate identification of biological indicators of disease progression and/or opportune therapeutic assessment.^5^ Although tissue biopsy and blood biomarker tests can serve as gold standards for disease diagnosis and pathological profiling (*e.g.*, in major organs like the liver, kidneys, *etc.*),^6^ the high invasiveness, non-real-time reporting, unsatisfactory precision and risk of complications are considerable defects in clinical practice.^7^ Alternatively, molecular imaging approaches such as ultrasonography (US), computed tomography (CT), magnetic resonance imaging (MRI) and optical imaging enable non-invasive and spatiotemporal mapping of pathophysiological changes in intact organisms;^8–13^ however, the lack of a valid method for the real-time monitoring of disease dynamics, and more importantly, punctual feedback of the therapeutic efficacy, remains a huge technical barrier, which vitally must be overcome for personalized and precision medicine.^14^

As essential biological messengers, reactive oxygen species (ROS) and their induced oxidative stress play significant roles in both biological signal transduction and the progression of pathologies ranging from cancers,^15,16^ and metabolic diseases,^17,18^ to cardiovascular,^19^ and even neurodegenerative disorders.^20,21^ Moreover, extensive investigations have witnessed the great potential of ROS as valuable biomarkers for effective disease diagnosis and feedback-informed therapy. In spite of the remarkable advances in ROS sensing techniques, accurate monitoring of various radical species dynamics, and more importantly, real-time correlation of these pervasively changing bio-indicators with disease progression and therapeutic response remain as bottlenecks. Recent studies have attempted to monitor dynamic ROS metabolism for the measurement of pathophysiological evolution by utilizing multispectral optoacoustic tomography (MSOT) and near-infrared (NIR) light-mediated nanotechnology;^22–28^ in particular, some oxidative stress-responsive photoacoustic or luminescent imaging platforms for oxidative pathological diagnosis have received tremendous attention.^29–37^ However, despite the initial success in disease sensing, relevant research focusing on dynamic manifestation of the oxidative stress status for non-invasive monitoring of the therapeutic response and beyond, especially in precise pharmaceutical intervention guidance in complex living conditions, remains a pressing and challenging goal, and relevant research has been rarely reported.

**Scheme 1:**
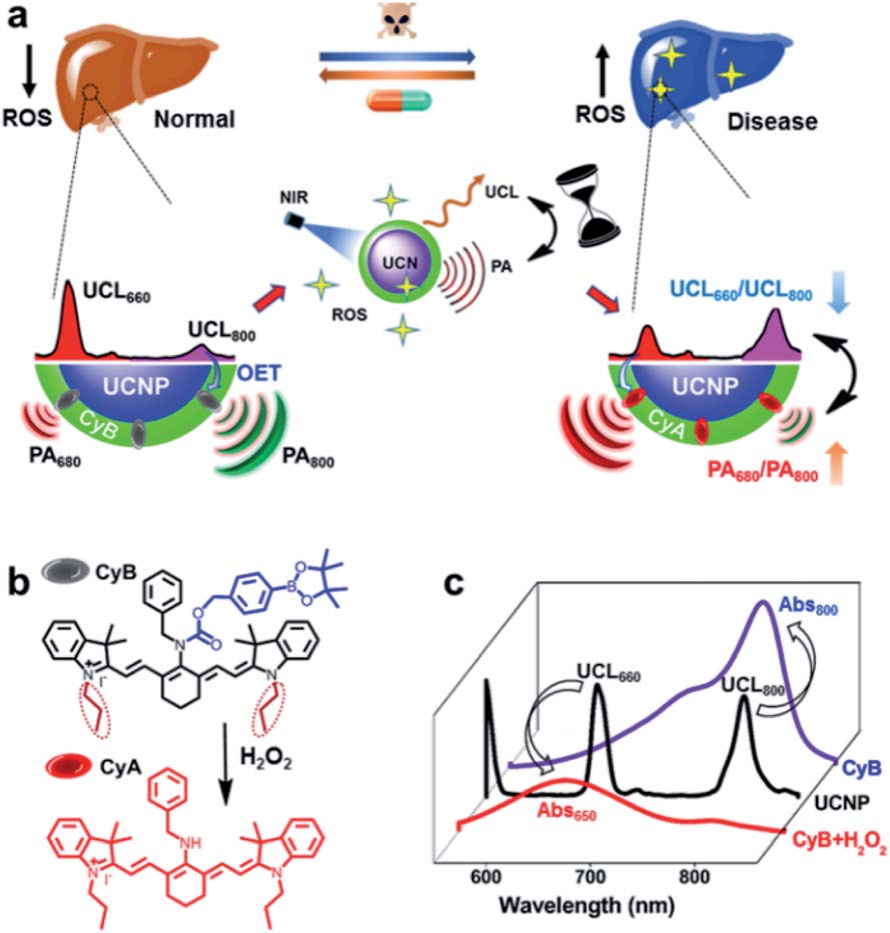
The principle of UCN design. (a) NIR nanoprobe-facilitated PA and UCL cross-referencing technique for simultaneous hepatopathological profiling and liver disease therapeutic assessment. (b) ROS responsive mechanism of the small-molecule probe, CyB. (c) Optical energy-transfer processes of the nanoprobe UCN before and after H_2_O_2_ stimulation.

Here, innovative nanoprobe-facilitated dual-modal imaging based on specific ROS activation is presented for precise evaluation of liver disease biology and dynamic therapeutic response assessment in living mice. Typically, we innovate such a cross-referencing imaging strategy by integration of MSOT with upconversion nanoparticle (UCNP)-mediated luminescence imaging, thereby taking advantage of the unique deep-tissue penetration, high spatiotemporal resolution and mutual corrective accuracy from both the MSOT and upconversion luminescence (UCL) imaging modalities.^38,39^ By tailoring the spectral overlaps between the multiplexing UCL response and the absorption shift of the H_2_O_2_-sensitive cyanine dye, this UCNP/cyanine-based nanoprobe (UCN) offers a convertible absorption blue-shift due to the chemo-specific reaction between ROS and the probe molecule that can be captured by MSOT. Meanwhile, the ROS-triggered response simultaneously induces multispectral luminescence changes, which exhibit the opposite trend of the UCL signal variation in comparison to the one observed in MSOT. Such spectrally opposite dual-modal imaging outcomes supply a ratiometric cross-referencing system that enables a full-package solution *via* the combination of the advantages of both optical full-field implementation and high-resolution photoacoustic tomography (PAT), thus allowing more sensitive and accurate evaluation of local disease biology and drug treatment responses, due to the self-calibrating feature of the reverse-ratiometric PA and UCL imaging technology (Scheme 1).

Compared with conventional therapeutic evaluation techniques like tissue biopsies and blood tests, our work not only effectively overcomes the limitations of invasiveness, discontinuity and hysteresis, but also exhibits superior sensitivity and spatiotemporal precision based on PA and UCL cross-referencing imaging, which greatly promotes the feasibility of opportune guidance for pharmaceutical intervention and new drug development.

## Results and discussion

### Design of the PA and UCL cross-referencing nanoprobe

Scheme 1a illustrates the integration of MSOT with UCL imaging by a cross-referencing ROS-sensitive nanoprobe, which offers a distinct paradigm for non-invasive and precise evaluation of disease pathological progression and assessment of the therapeutic response. Given that liver disease is of great harm and is known as a major killer of the population at different ages,^4,40^ we chose acute liver failure as the disease model to prove our concept. So far, extensive studies clearly indicate the correlation of cumulative ROS production with induced cellular redox imbalance, oxidative damage and liver dysfunction.^17,18^ Therefore, real-time monitoring of oxidative stress status in liver damage progression could contribute to the early identification of diseases and pathological profiling, as well as effective diagnostics and treatment evaluation in clinical settings.

The rationale for our nanoprobe design is mainly based on the principle that the ROS-sensitive chromogenic-convertible cyanine molecule acts as a specific PA reporter; meanwhile, ROS triggered reaction can also lead to UCL responses due to the optical energy-transfer (OET) between robust multi-emissive UCNPs and cyanine chromophores. Typically, NIR-responsive UCNPs with maximum emission (*λ*_UCL_) at 800 and 660 nm and cyanine dye molecules (CyB) are combined into one unified nanoplatform. Upon H_2_O_2_ stimulation, the electron-withdrawing boronate-substituted CyB can be specifically converted to its precursor structure, CyA, which triggers a hypsochromic shift from 800 to 650 nm (Scheme 1b and c).^41^ More importantly, the desirable spectral overlaps between UCL and the absorption band of either CyB or CyA (Scheme 1c)^42^ enable a decreased ratio of UCL_660_/UCL_800_ but an opposite signal change of PA_680_/PA_800_. Such an interlocking strategy can internally cross-reference two individual modalities, and permits high spatiotemporal and quantitative precision for H_2_O_2_ analysis, which therefore provides an omnidirectional solution for systemic manifestation of dynamic oxidation variation in disease progression and drug treatment.

### Fabrication and characterization of the UCN

The cyanine molecule CyB was synthesized (Scheme S1†) through a Cy7-based starting framework (IR780)^41^ but contains propyl substitution to reach a red-shifted absorption at ∼800 nm. To achieve an effective photon upconversion, the core–shell UCNPs (NaGdF_4_:Yb/Tm/Er@NaGdF_4_) were prepared by the solvothermal method (Scheme S2†),^11,42,43^ and the modulation of Tm^3+^ and Er^3+^ lanthanide elements enables multicolor emissions (Fig. S1†, *λ*_UCL_ = 800, 660 nm *etc.*) that well match the absorption peak of either CyB or CyA. The oleic acid (OA)-coated UCNPs were further functionalized with branched polyethylenimine (PEI) and polyethylene glycol (PEG) to improve the biocompatibility, which also facilitates the physical encapsulation of hydrophobic chromophore CyB into the inner hydrophobic spaces on the surface of the UCNPs.^44^ The CyB loading efficacy in the UCN was determined to be 4.84% from the absorbance at 800 nm (Fig. S2†), and the persistent absorbance indicates a good loading stability where the UCN was kept in HEPES buffer with or without 10% FBS (Fig. S3†). The typical transmission electron microscopy (TEM) images in Fig. 2a and S4† show a spherical morphology of the bare UCNPs and the nanoprobe, respectively. The average hydrodynamic diameter of the nanoprobe was further determined to be ∼78 nm.

### ROS response of the UCN

**Fig. 1:**
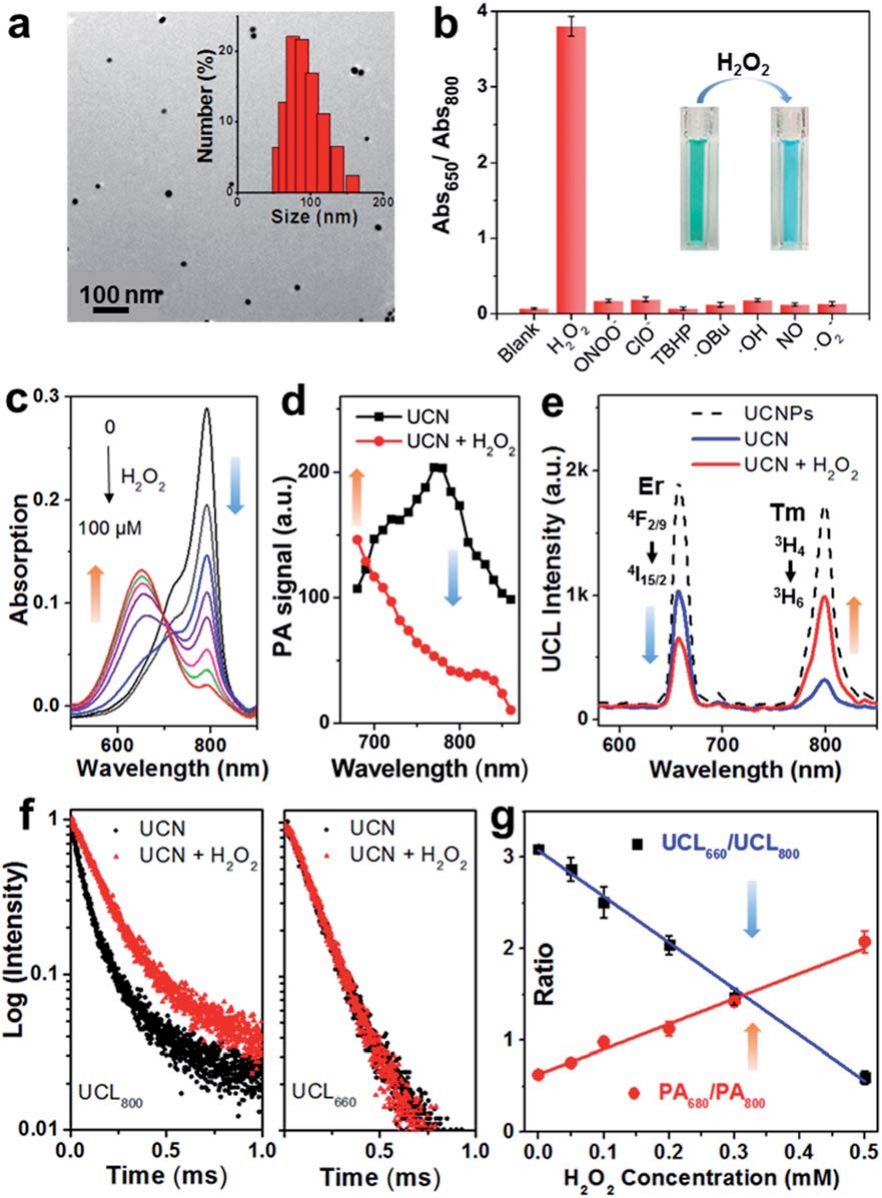
The characterization and ROS response of the UCN in buffer. (a) TEM image and hydrodynamic diameter distribution of the as-prepared UCN. (b) The specificity of the UCN response towards different ROS: H_2_O_2_, peroxynitrite (ONOO^−^), hypochlorite (^−^OCl), *tert*-butyl hydroperoxide (TBHP), *tert*-butoxy radicals (˙OtBu), hydroxyl radicals (˙OH), nitric oxide (NO) and superoxide radicals (˙O^2−^). (c) Absorption spectral changes of the UCN (0.1 mg mL^−1^) upon incubation with H_2_O_2_ from 0 to 100 μM. (d) The PA response of the UCN (0.5 mg mL^−1^) with 100 μM H_2_O_2_. (e) UCL responses of 0.5 mg mL^−1^ UCNPs, the UCN and 100 μM H_2_O_2_-treated UCN, respectively (Ex. 980 nm). (f) UCL_800_ and UCL_660_ lifetime spectra of label-free UCNPs, nanoprobe UCN and H_2_O_2_-treated UCN, respectively (Ex. 980 nm). (g) PA (red) and UCL (blue) ratiometric cross-referencing for H_2_O_2_ detection.

Firstly, the sensing specificity was evaluated by incubation of the UCN with various radical species. As shown in Fig. 1b, only the H_2_O_2_ incubated group shows the highest Abs_650_/Abs_800_ ratio (∼3.8) due to the absorption blue-shift triggered by chemo-specific cleavage of the caged boronate group; in contrast, no significant ratio change (<0.2) could be found in the groups incubated with any other type of ROS, indicating the superior selectivity of the nanoprobe to H_2_O_2_. Moreover, the absorption spectra of the UCN towards different concentrations of H_2_O_2_ can be observed in Fig. 1c with a good linear correlation. The kinetics of the H_2_O_2_-induced absorbance changes from the nanoprobe (at 650 and 800 nm) were investigated, which demonstrated a clear ratio increase of Abs_650_/Abs_800_ and nearly 80% completion of probe activation within 10 min (Fig. S5†). Remarkably, the PA signal change, upon response to H_2_O_2_, exhibited a similar trend that the PA_800_ intensity is significantly decreased, while only a slight enhancement of PA_680_ can be found (Fig. 1d). This dual-wavelength PA response can thus provide the possibility of ratiometric PA imaging of H_2_O_2_. Interestingly, compared with the PA results, the radical triggered UCL spectra changes (Fig. 1e) present similar ROS specificity, but show the opposite trend that UCL_800_ is greatly increased, whereas UCL_660_ is quenched slightly, due to the OET process between the UCNPs and the convertible small-molecule dyes (CyB or CyA). This further indicates that our nanoprobe could also work for ratiometric UCL detection of H_2_O_2_.

Both steady-state UCN luminescence and dynamic luminescence lifetime measurements validate the UCL energy transfer for H_2_O_2_ sensing. As shown in Fig. 1e, the UCL intensity at 800 nm (blue curve) is significantly quenched as compared to the label-free UCNPs (black dashed curve) with the OET efficiency of ∼81%.^42^ However, upon H_2_O_2_ reaction (red curve), the OET efficiency of UCL_800_ decreases approximately to 40%, while the OET value of UCL_660_ is found to enhance from 46% to 68%. Likewise, the luminescence lifetimes at 800 and 660 nm show similar trends. Compared with label-free UCNPs, the UCL_800_ lifetime of the CyB-encapsulated nanoprobe decreases from 202 to 74 μs (Fig. S6†), mostly attributed to the process of energy transfer between the UCNPs and the loaded convertible dyes. Besides, the H_2_O_2_-induced UCN lifetime variations (Fig. 1f) demonstrate the significant enhancement of UCL_800_ (from 74 to 169 μs), while only very slight change of UCL_660_ is observed (140 and 145 μs). Such dual-wavelength luminescence changes provide a ratiometric analysis to facilitate precise manifestation of dynamic oxidative status through the as-prepared nanoprobe.

Then, the capability of the nanoprobe for ratiometric H_2_O_2_ detection was investigated by PA and UCL modalities. The results in Fig. 1g reveal good linear correlations but opposite ratio slopes in both PA_680_/PA_800_ and UCL_660_/UCL_800_, as a function of detection (LODs) of PA and UCL was determined to be ∼0.1 and 0.07 μM respectively, suggesting that the nanoprobe is sensitive enough to detect biological H_2_O_2_ at concentrations as low as the submicromolar level. In line with the good sensitivity and quantitative capability for H_2_O_2_ sensing, this nanoprobe demonstrated opposite signal changes between PA_680_/PA_800_ and UCL_660_/UCL_800_, thus offering a novel cross-referencing strategy to orthogonally correlate the PA and UCL techniques, greatly minimizing the pseudo signal response to guarantee the precise validation of abnormal oxidation under highly complex and dynamic living conditions.

### Cross-referencing of PA and UCL for endogenous H_2_O_2_ detection

The potential of UCN for endogenous H_2_O_2_ sensing was further investigated in murine RAW264.7 macrophage cells by both PA and UCL imaging. Briefly, excessive cellular ROS production was achieved by treatment of the cells with lipopolysaccharide (LPS), a commonly used bacterial endotoxin. Upon 4 h of LPS stimulation and nanoprobe incubation (0.1 mg mL^−1^), both living cells and cell lysates were collected for confocal imaging and MSOT analysis.

**Fig. 2:**
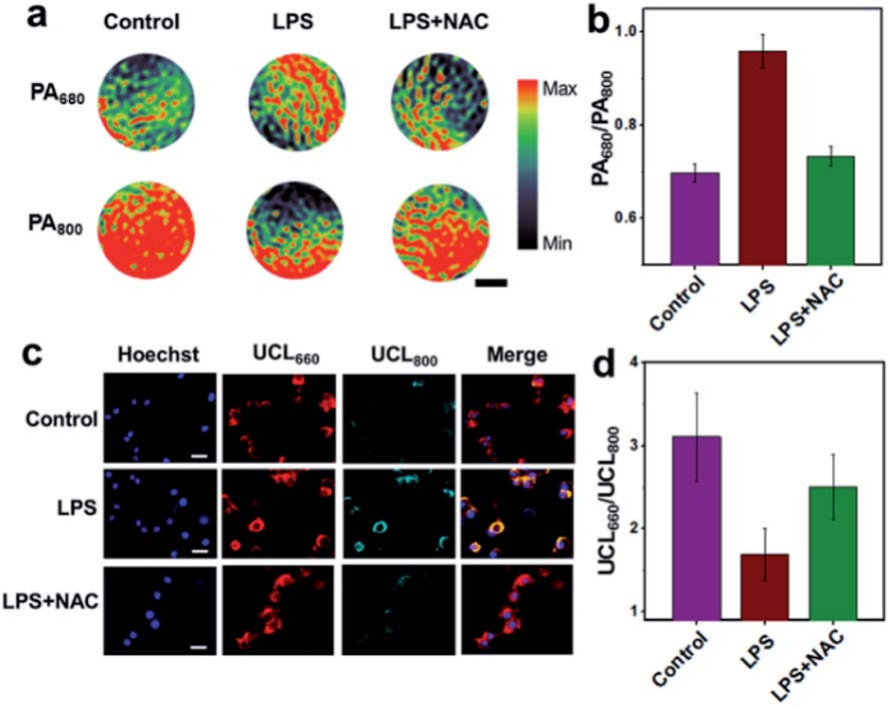
Endogenous H_2_O_2_ detection in RAW264.7 cells by UCN. (a) PA images of cell lysates incubated with 0.1 mg mL^−1^ UCN in the absence/presence of LPS (2 μg mL^−1^) or the ROS scavenger NAC (0.3 mM), respectively. Scale bar: 1 mm. (b) The plot of PA_680_/PA_800_ ratios in different groups. Data were represented as mean ± SD. (c) UCL imaging of RAW264.7 cells treated with UCN in different groups as above. Hoechst (blue, Ex: 405 nm, Em: 460/50 nm), UCL_660_ (red, Ex: 980 nm, Em: 640/50 nm), UCL_800_ (cyan, Ex: 980 nm, Em: 790/30 nm). Scale bar: 50 μm. (d) The plot of UCL_660_/UCL_800_ ratios in different groups. Data were analyzed by ImageJ and represented as mean ± SD.

As shown in Fig. 2a, the PA signal at 680 nm for the LPS-treated cell lysates is much higher than the control without LPS as well as the group pretreated with *N*-acetylcysteine (NAC, a ROS scavenger), while the PA_800_ intensity in the LPS-stimulated group exhibits an obvious attenuation as compared to other normal cells. Furthermore, the ratio of PA_680_/PA_800_ in the LPS-treated group is determined to be ∼0.96, whereas a ratio of only ∼0.70 can be found in the control group (Fig. 2b and S7†), suggesting a positive PA ratiometric change triggered by the endogenous H_2_O_2_. In contrast, the UCL imaging shows different trends of luminescence ratio changes (Fig. 2c). Without LPS, intracellular red emission (UCL_660_) is clearly detected, with the UCL_800_ emission (cyan) particularly weak due to the OET process. As expected, an obvious UCL_800_ signal can be found upon LPS stimulation, indicating a reduced energy transfer between the UCNPs and CyB, probably owing to the absorption band blue-shift induced by H_2_O_2_ reaction. Although similar UCL_660_ signals are observed in different groups, the ratiometric UCL_660_/UCL_800_ values in Fig. 2d reflect an obvious difference, in which the ratio of the LPS-treated group (∼1.7) is significantly lower than those observed in the control (∼3.1) and NAC-pretreated cells (∼2.5). In addition, further cell viability studies in RAW264.7 and Kupffer cells indicate the negligible cytotoxicity of the UCN after 24 h of co-incubation (Fig. S8†). These results demonstratively prove that our nanoprobe is feasible for cellular H_2_O_2_ sensing by both PA and UCL imaging, and more importantly, such a cross-referencing strategy based on reversed ratiometric PA_680_/PA_800_ and UCL_660_/UCL_800_ holds superior sensitivity and accuracy for endogenous H_2_O_2_ detection, which may provide robust applicability in complex living conditions.

### *In vivo* dynamic cross-referencing manifestation of radical stress induced liver disease biology

As a major metabolic and immunological organ, the liver serves various important functions including energy metabolism, storage and poison excretion.^45^ However, a variety of genetic or acquired factors such as viruses, bacteria, drugs and alcohol usage that can disturb liver functions and lead to different types of disorders,^46–48^ which engender substantial impacts on public health.^4,49^ In light of the close-knit interplay between dynamic redox imbalance and hepatic diseases,^50,51^ it is highly essential to investigate the detailed mechanisms of hepatopathological progression by using our nanoprobe.

We firstly evaluated the *in vivo* biodistribution and biosafety of our nanoprobe *via* intravenous (i.v.) injection of the UCN (5 mg mL^−1^, 100 μL) into mice and monitored the signals with MSOT and UCL imaging. As shown in Fig. S9a–c†, *in vivo* and *ex vivo* UCL imaging confirm the higher accumulation of the UCN in the liver than the other main organs, and the nanoprobe could be cleared out after 24 h. The obvious liver localization of the UCN was also validated *via* PA_800_ imaging in Fig. S10a,† and the consistent PA_800_ signals further indicate the good biostability of the loaded dye on the UCN surface. Moreover, the potential biosafety issue of the UCN was studied by biochemical serum assay and histological analysis. The results reveal negligible toxicity towards mice livers and other tissues (*e.g.* heart, lungs, liver, spleen and kidneys *etc.*) after UCN administration (Fig. S10b and c†), further demonstrating the good biocompatibility and feasibility of our nanoprobe for *in vivo* liver sensing applications.

So far, the first-line antituberculotic drug, isoniazid (INH)-induced hepatotoxicity has been proposed to be closely linked with oxidative stress; however, exact mechanisms still need to be identified.^52^ In this study, we established an acute liver failure model in Balb/c nude mice (*n* = 5) *via* intraperitoneal injection (i.p.) of a toxic dose of INH (100 mg kg^−1^). Inspired by the reliable biodistribution results aforementioned, the liver accumulation of the UCN reaches a plateau from 1 to 3 h after *i.v.* injection, suggesting a good dynamic imaging window with less interference caused by the nanoprobe metabolism *in vivo*. Thereby, time-resolved PA and UCL imaging of INH-pretreated and normal mice was performed 1 h post UCN (5 mg mL^−1^, 100 μL) administration to explore the complications of INH-induced oxidative stress in liver pathological progression.

**Fig. 3:**
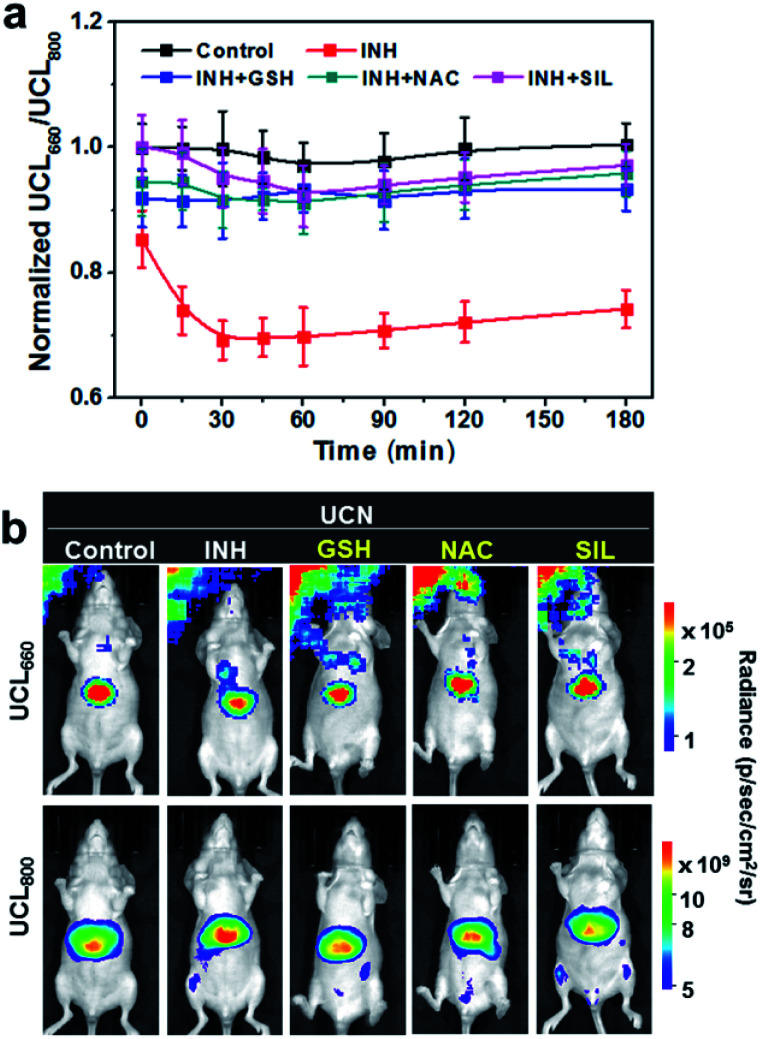
*In vivo* ratiometric UCL imaging. (a) Dynamic ratios of UCL_660_/UCL_800_ at various time points in UCN-pretreated (5 mg mL^−1^, 100 μL) mice upon INH (100 mg kg^−1^) stimulation with or without antioxidant therapeutic reagents (*e.g.*, GSH, NAC and SIL, 200 mg kg^−1^, respectively). Ratiometric values represent means ± SD (*n* = 5). (b) UCL images at 30 min in INH-treated or hepatoprotective drug-pretreated mice upon UCN injection.

The dynamic luminescence signal changes of UCL_660_ and UCL_800_ were determined in the liver with different time intervals. The intensities of UCL_800_ in INH-treated mice are gradually enhanced in comparison to the control mice without INH treatment, while similar UCL_660_ signals can be detected in both groups within the imaging period (Fig. S11†). The ratiometric UCL_660_/UCL_800_ values in Fig. 3a obviously show a remarkable downward trend in the mice with INH stimulation. Moreover, the dynamic ratiometric imaging clearly indicates the oxidative burst induced by INH within 30 min with a subsequent trend of UCL_660_/UCL_800_ declining. As indicated in Fig. 3b, there is a much higher UCL_800_ intensity observed at 30 min, and only a slight decrease of UCL_660_ is detected in INH-treated mice when compared to the mice in the control group. These results demonstrate the great potential of our nanoprobe for ratiometric screening of oxidation dynamics in living animals.

**Fig. 4:**
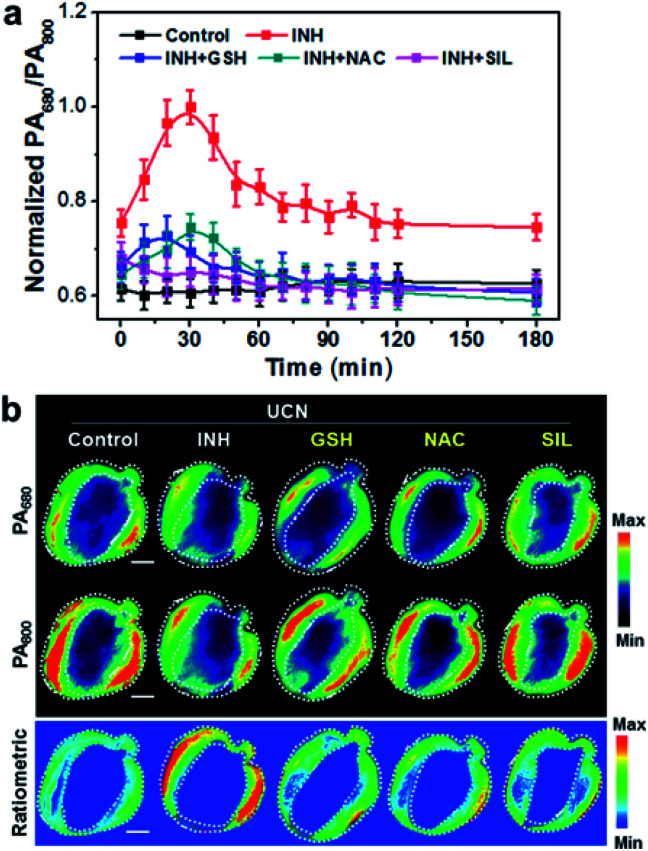
*In vivo* ratiometric PA imaging. (a) Dynamic ratios of PA_680_/PA_800_ at various time points in UCN-pretreated (5 mg mL^−1^, 100 μL) mice upon INH (100 mg kg^−1^) stimulation with or without antioxidant drugs (*e.g*., GSH, NAC and SIL, 200 mg kg^−1^). Ratiometric values represent means ± SD (*n* = 5). (b) PA images at 30 min in INH-treated or drug-pretreated mice upon UCN injection. The region of interest (ROI) indicates the PA signal in the liver area from the cross section z-stack orthogonal maximal intensity projection (MIP) images. Scale bars: 1 cm.

To validate the applicability of photoacoustic imaging *in vivo*, the cross-sectional MSOT images covering the whole liver were captured at a 10 min interval in normal mice and animals treated with hepatoxic INH 1 h after UCN administration. The PA signal changes at 680 and 800 nm were monitored and quantified (Fig. S12†). Interestingly, the dynamic changes of both the PA_680_ and PA_800_ signals show the opposite trends in comparison to UCL imaging. Typically, the PA_800_ intensity in the INH-treated mice displays a noticeable reduction in the initial 30 min and then subsequently rises over the time duration, most likely due to the ROS response and dynamic metabolism of the UCN in the liver. Consequently, the ratiometric variation of PA_680_/PA_800_ in Fig. 4a exhibits an upward trend with the peak value at ∼30 min in the INH-treated group; such a ratiometric change is exactly opposite to that of UCL_660_/UCL_800_, strongly suggesting that the cross-referencing feature of PA and UCL is capable of *in vivo* ROS imaging. Additionally, we also constructed the pseudo-color PA images and analyzed the cross-section anatomy of the liver (Fig. 4b). The increased PA_680_/PA_800_ (blue to red) can be visualized between normal and liver-injured mice treated with INH, clearly demonstrating the feasibility of the UCN for precise oxidative stress screening *in vivo*.

In line with the great potential of UCNs, our nanoprobe successfully builds a bridge between MSOT and UCL imaging for spatiotemporal sensing of ROS dynamics *in vivo*. More importantly, the internal ratiometric cross-referencing of the PA and UCL signals enables much higher accuracy for real-time manifestation of oxidative damage in drug-induced liver pathological progression, which can greatly facilitate further investigations into noninvasive pharmacological assessment.

### *In vivo* dynamic cross-referencing assessment of therapeutic responses

Inspired by the promising results in real-time monitoring of ROS-induced hepatotoxicity *in vivo*, we further studied the potential of the UCN for dynamic assessments of liver treatment feedback. To this end, three clinically used drugs, including glutathione (GSH), *N*-acetylcysteine (NAC) and silibinin (SIL), that can effectively trigger the antioxidant defense and cure various types of drug-induced liver injury (DILI) were selected to evaluate the applicability of the UCN. Generally, MSOT and UCL imaging were performed in INH-treated mice administrated with the UCN. The DILI treatment effects based on drug molecules of GSH (200 mg kg^−1^, i.v.), NAC (200 mg kg^−1^, i.p.) and SIL (200 mg kg^−1^, i.g.) were systematically evaluated through MSOT and UCL analysis.^53–55^ As expected, all these drugs definitely play roles in the inhibition of liver injury by suppressing the ROS overproduction induced from hepatotoxic INH treatment, which can be noninvasively and sensitively reflected by integrated PA and UCL imaging. As shown in Fig. 3, a significant signal enhancement in UCL_660_/UCL_800_ can be observed, whereas there is an obvious ratio decrease for PA_680_/PA_800_ (Fig. 4) in comparison to INH-stimulated hepatotoxic mice. Moreover, representative PA and UCL images of drug-treated mice show similar signals within short time period therapy (∼30 min) to those observed in normal mice, suggesting relatively low oxidative stress in the liver after drug treatment. Notably, slightly progressive oxidation could still be observed after INH injection within ∼1 h in the mice treated with different liver protective drugs. Among the therapeutic assessments, it is easy to find that the SIL drug molecule exhibits superior efficacy to curb liver ROS generation caused by INH metabolism, and the antioxidant GSH exhibits slightly better action than that of NAC against the early stage of liver injury (<1 h), while almost identical hepatoprotection can be detected after prolonged INH stimulation (1–3 h). Such manifestations enable the horizontal comparison of different therapeutic effects, which is of great importance in practical high-throughput drug screening.

### Comparison with conventional liver function tests

**Fig. 5:**
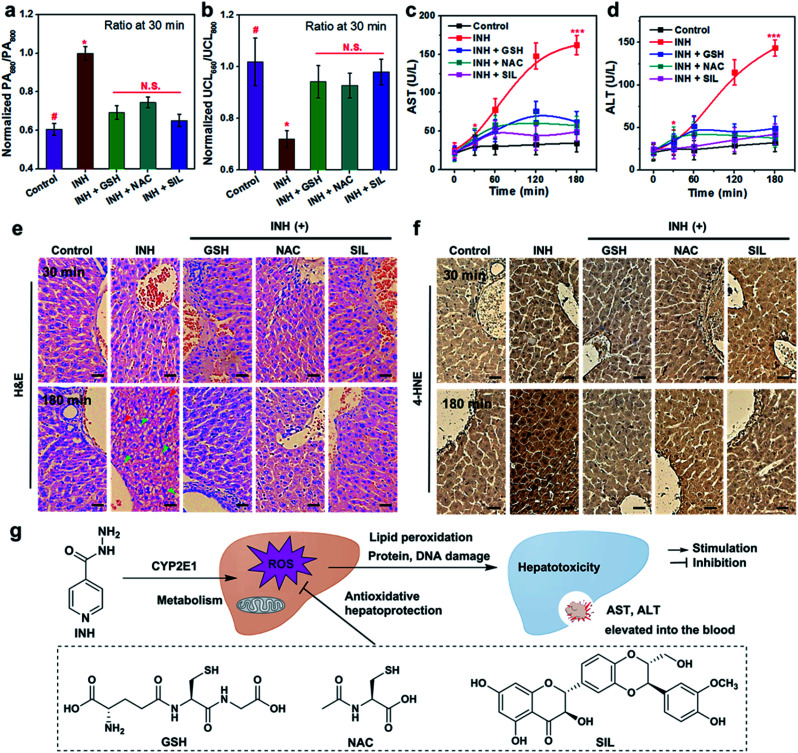
Multiplexed profiling of liver pathological alternations and comparative assessments of therapeutic responses. (a and b) PA_680_/PA_800_ and UCL_660_/UCL_800_ values at 30 min in the liver for the INH-treated or antioxidant drug (GSH, NAC and SIL)-pretreated mice upon UCN injection. Columns represent means ± SD (*n* = 5). # indicates the control group. (c and d) Serum aspartate transaminase (AST) and alanine aminotransferase (ALT) levels for the mice upon treatment with INH or hepatoprotective drugs at different times. Data represent means ± SD (*n* = 5). (e) H&E staining of liver tissues at 30 and 180 min after INH treatment with or without drugs (*n* = 5). Arrowheads mark centrilobular vein fibrosis (blue), swollen hepatocytes (green), and inflammatory infiltration (red), respectively. CV: central vein. Scale bars: 50 μm. (f) Immunohistochemical study of 4-hydroxynonenal (4-HNE) staining in liver sections at 30 and 180 min after INH treatment with or without drug molecules (*n* = 5). Black arrowheads mark 4-HNE-positive lesions. Scale bars: 50 μm. (g) Illustration of INH-induced liver dysfunction and antioxidant drug treatment effects. The *p*-values (*p < 0.05, ***p < 0.001) were determined by *t*-test.

To further validate the reliability of UCN-based therapeutic response assessments, the commonly used biological assays including serum transaminase tests and liver histological studies were carried out to correlate the dynamic oxidative implications with the different disease stages, and the dynamic treatment responses were systematically verified by our PA and UCL cross-referencing nanoprobe. As shown in Fig. 3 and 4, both PA and UCL imaging exhibit obvious signal variations in INH-treated hepatotoxic mice pretreated with antioxidant drugs as compared to those of control mice. In particular, the most significant upregulation of PA_680_/PA_800_ can be easily observed, while the simultaneous downregulation response of UCL_660_/UCL_800_ can be detected within 30 min (Fig. 5a and b), clearly indicating that the early oxidative stress burst in the liver injuring process could be distinctly manifested by the UCN.

Although the gold-standard liver function tests (LFTs) through the amount of aspartate transaminase (AST) and alanine aminotransferase (ALT) (Fig. 5c and d) demonstrate tangible evidence of time-dependent liver damage triggered by INH, the administration of GSH, NAC and SIL can remarkably suppress or even reverse the pathological progression. It should be noted that such serum biochemical assays have difficulty in differentiating hepatotoxic responses at the initial stage (*e.g.* <30 min). Furthermore, liver histological studies with hematoxylin & eosin (H&E) staining indicate no morphological changes observed in liver tissues at an earlier stage (*e.g.* 30 min) (Fig. 5e), mainly due to a low degree of substantial damage. Obvious swollen hepatocytes and inflammatory infiltration can only be found at the earliest 3 h post INH stimulation. Furthermore, since oxidative stress plays crucial roles in overdosed INH causing acute liver failure, an immunohistochemical study based on 4-hydroxynonenal (4-HNE) was utilized to investigate the possibility of lipid peroxidation (LPO) occurring in the damaged liver. As shown in Fig. 5f, prolonged INH stimulation (*e.g.* ∼3 h) significantly induces 4-HNE positive foci; meanwhile, the liver tissues with GSH, NAC or SIL pretreatment demonstrate negligible LPO lesions, clearly indicating their promising antioxidant effects. Similarly to the serum AST/ALT tests, LPO-associated histochemical analysis is difficult to use in effectively monitoring the dynamic progression of liver pathology and related therapeutic responses, especially at an early stage. Moreover, nanoprobe treatment alone will not induce any liver histological changes or LPO positive lesions (Fig. S13†), further revealing the reliable biosafety of the UCN for noninvasive PA and UCL imaging.

Apparently, although the serum tests and histological analysis are capable of diagnosing substantive liver injury and overall pharmacological actions, these methods are heavily limited in characterizing the incipient hepatotoxic response and dynamically monitoring the therapeutic effects, owing to the complicated sampling and testing processes. In contrast, UCN-assisted PA and UCL imaging display higher sensitivity and real-time capability for precise detection of dynamic liver pathogenesis and early therapeutic effects, without the unpleasant operations, hysteretic reporting and risk of complications usually suffered in clinical blood tests and liver biopsies. Most critically, such a unique technique offers a cross-referencing profile *via* reverse-ratiometry of PA_680_/PA_800_ and UCL_660_/UCL_800_ during the therapeutic assessments against liver dysfunction, which greatly enhances the sensing precision in complex conditions, as well as providing archetypal research to boost its applications in broader oxidative stress or even antioxidant pharmacological profiling, beyond liver disease (Fig. 5g). Other than the great merits for the applicability of the UCN, the concept of cross-referencing sensing also paves a new way to design highly accurate and versatile bioimaging probes.

## Conclusions

In summary, we introduce an innovative nanoprobe-facilitated cross-referencing molecular imaging technique through ROS triggered PA and UCL responses for reversed-ratiometric and noninvasive monitoring of oxidative dynamics. Such a unique system further promotes precise recognition of the complex interplay between oxidative stress status and liver pathological evolution, as well as being useful in the identification of local pathological progression for dynamic therapeutic feedback. Owing to the superb diagnostic performance compared to conventional blood tests and histological analysis, we envision that our study may serve as an archetypal system to encourage novel diagnostics not only for precise therapeutic assessment, but also to boost its applications in a brand new field of real-time pharmacological screening, especially in feedback-informed intervention guidance and new drug development.

## Ethical statement

This study was performed in strict accordance with the national guidelines for the care and use of laboratory animals (Certificate No. 20020008, Grade II) and was approved by the Institutional Animal Care and Use Committee (IUCAC) of the Soochow University Laboratory Animal Center (Suzhou, China).

## Conflicts of interest

The authors declare no conflict of interest.

## Supplementary Material

SC-011-C9SC04909F-s001
