# Supplementary material for: NIR nanoprobe-facilitated cross-referencing manifestation of local disease biology for dynamic therapeutic response assessment
Source: Chem Sci. 2019 Nov 27;11(3):803–11. doi: 10.1039/c9sc04909f (PMC8146619; doi:10.1039/c9sc04909f)
Supplement: SC-011-C9SC04909F-s001 [file SC-011-C9SC04909F-s001.pdf]

Electronic Supplementary Material (ESI) for Chemical Science.
This journal is © The Royal Society of Chemistry 2019

Supporting Information

NIR nanoprobe facilitated cross-referencing manifestation of local disease biology for dynamic therapeutic response assessment

Zhimin Wang[a‡], Xiangzhao Ai[a‡], Zhijun Zhang[a], Yong Wang[b], Xiangyang Wu[a], Richard Haindl[c], Edwin K. L. Yeow[a], Wolfgang Drexler[c], Mingyuan Gao[b], Bengang Xing[a*]

[a] Division of Chemistry and Biological Chemistry, School of Physical & Mathematical Sciences, Nanyang Technological University, 637371, Singapore.

[b] Center for Molecular Imaging and Nuclear Medicine, School for Radiological and Interdisciplinary Sciences (RAD-X), Soochow University, Suzhou 215123, China.

[c] Center for Medical Physics and Biomedical Engineering, Medical University of Vienna, 1090 Vienna, Austria.

Corresponding Author:

* E-mail: bengang@ntu.edu.sg

‡ These authors contributed equally.

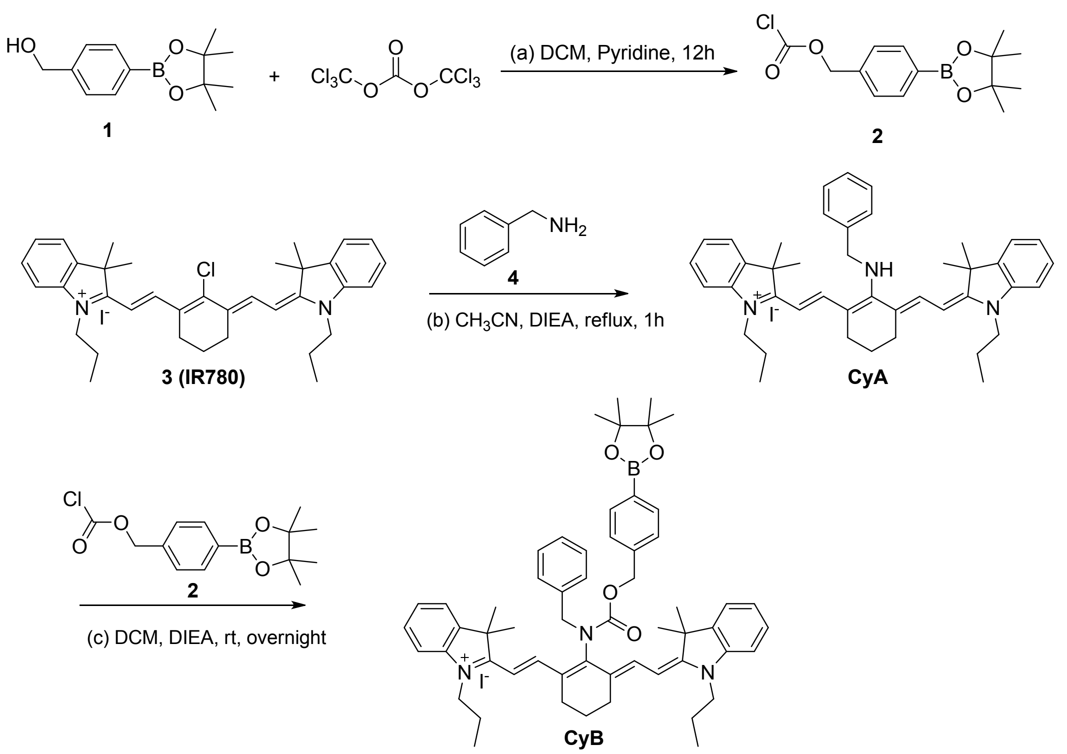

Scheme S1. Synthesis of ROS responsive probe CyB.

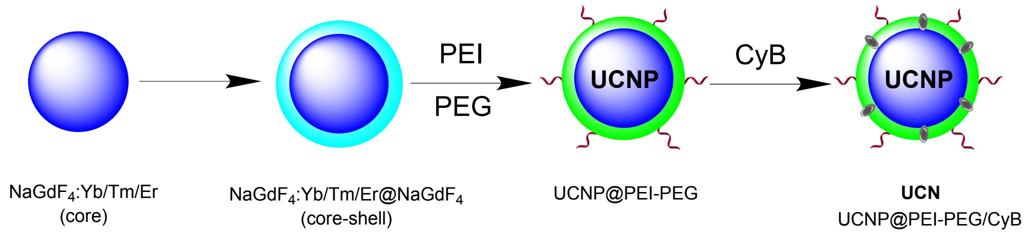

Scheme S2. Preparation of the nanoprobe UCN (UCNP@PEI-PEG/CyB).

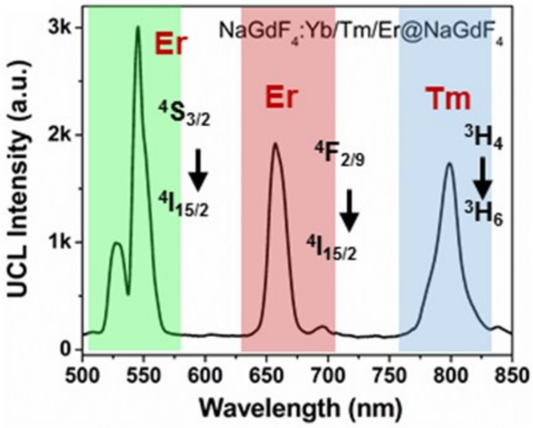

Figure S1. Upconversion luminescence (UCL) spectra of the core-shell UCNPs under 980 nm laser excitation.

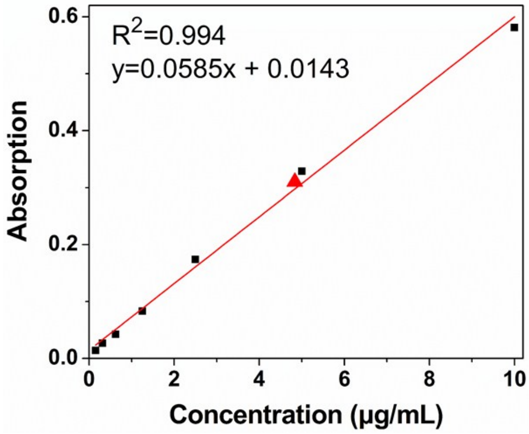

Figure S2. Absorbance at 800 nm as a function of CyB with different concentrations (0.0156 - 10 μg/mL). The CyB loading content (red symbol) in UCN (0.1 mg/mL) was determined to be 4.84 %.

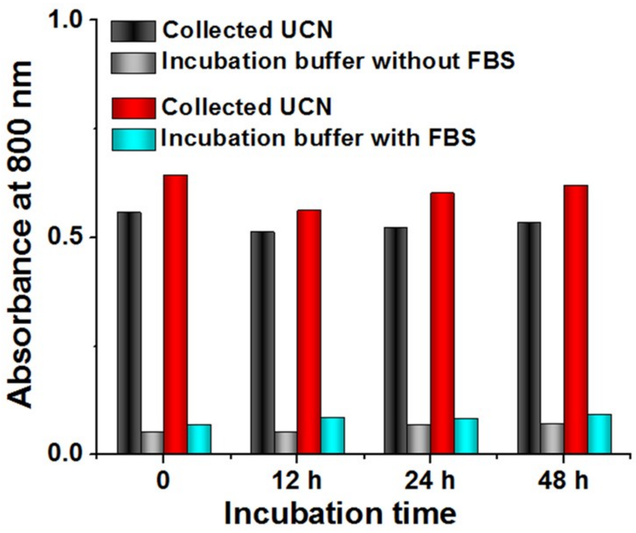

Figure S3. Absorption of collected UCN (0.5 mg mL^{-1}) and incubation buffer (HEPES, 10 mM, pH = 7.4) without or with 10% FBS at different time points (0, 12, 24, 48 h).

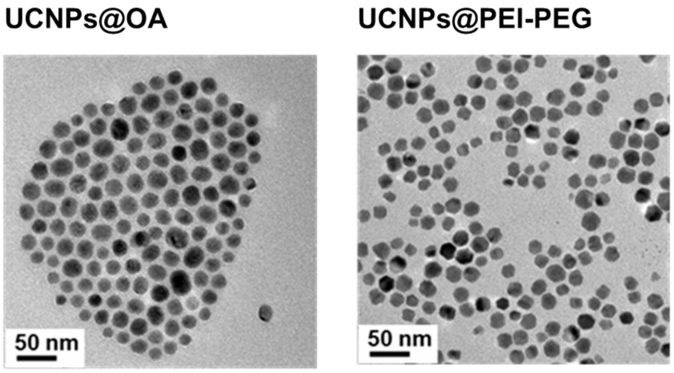

Figure S4. TEM images of oleic acid (OA)-coated core-shell UCNPs and further modified UCNP@PEI-PEG.

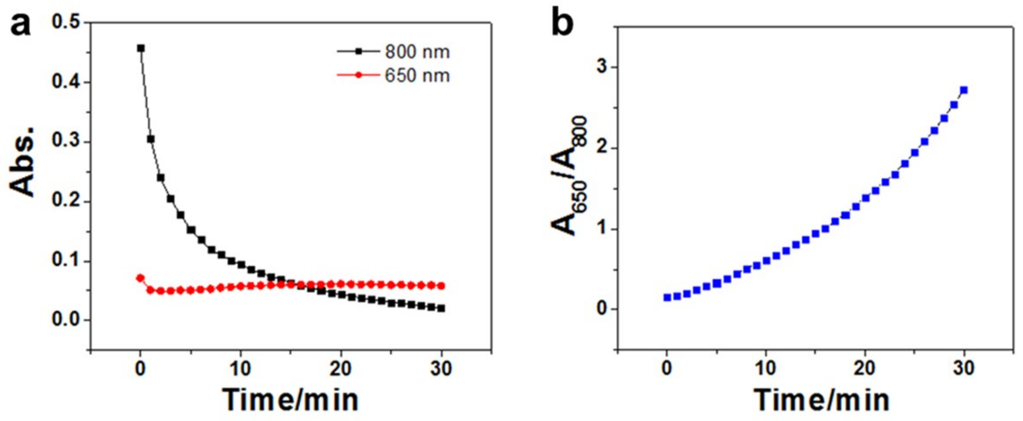

Figure S5. The kinetics of the nanoprobe, UCN (0.1 mg/mL) in the presence of 100 μM H_2O_2.

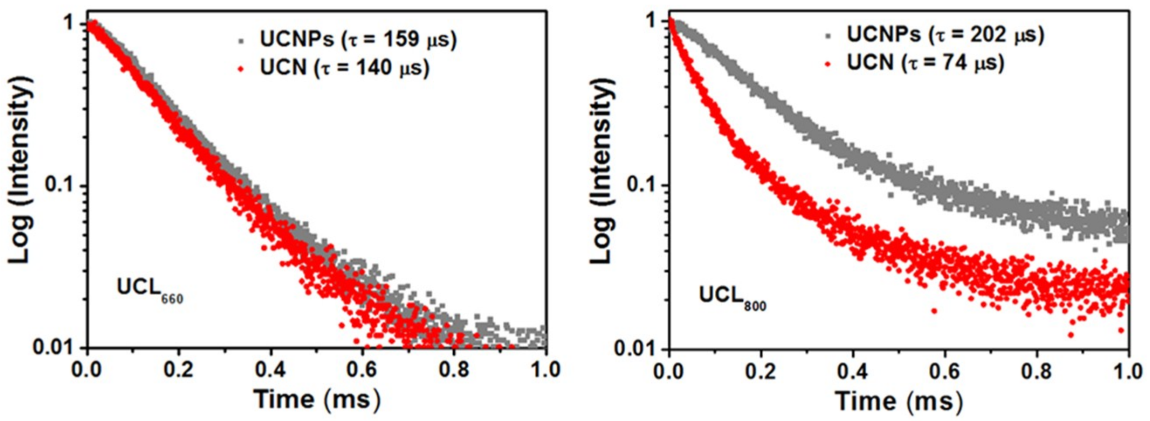

Figure S6. The lifetime spectra for UCL_{660} and UCL_{800} of UCNPs with or without CyB encapsulation on particle surface (UCN). Ex. 980 nm.

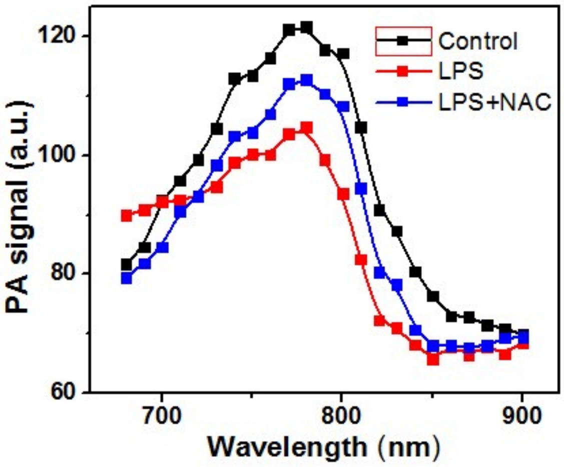

Figure S7. PA signals of cell lysates incubated with 0.1 mg/mL UCN in the absence or presence of the ROS stimulant LPS (2 μg/mL) or the ROS scavenger NAC (0.3 mM), respectively.

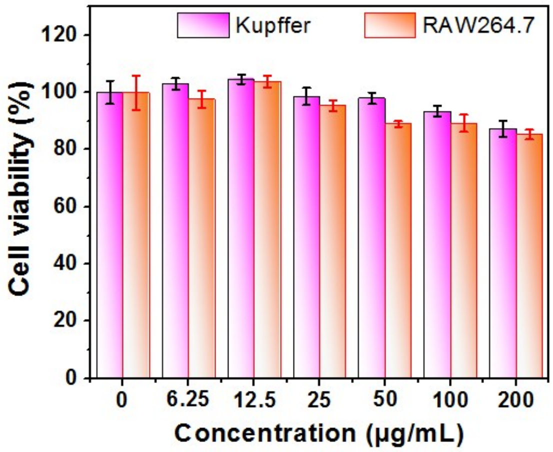

Figure S8. The viability of RAW264.1 and Kupffer cells after 24 h incubation with different concentration of the nanoprobe, UCN.

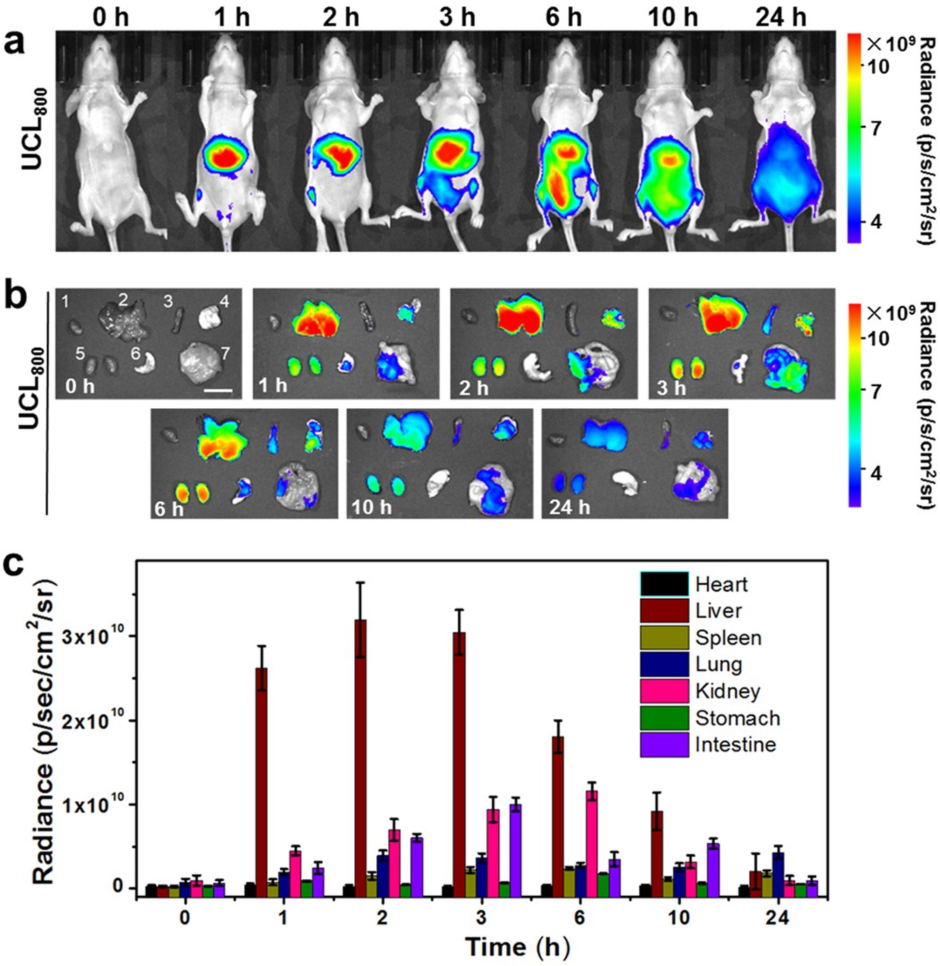

Figure S9. *In vivo* dynamic biodistribution of UCN. (a) UCL imaging of mice after tail-vein injection of the nanoprobe (5 mg/mL, 100 μL) at different time points. UCL_{800} signals (Em: 790/30 nm) were recorded upon 980 nm laser excitation. (b) *Ex vivo* UCL_{800} imaging of different organs post UCN injection at different times. Scale bar: 1 cm. Organs from 1-7: 1, Heart; 2, Liver; 3, Spleen; 4, Lung; 5, Kidney; 6, Stomach; 7, Intestine. (c) Quantification of UCL_{800} signals in different organs at different times. Data were represented as mean ± SD.

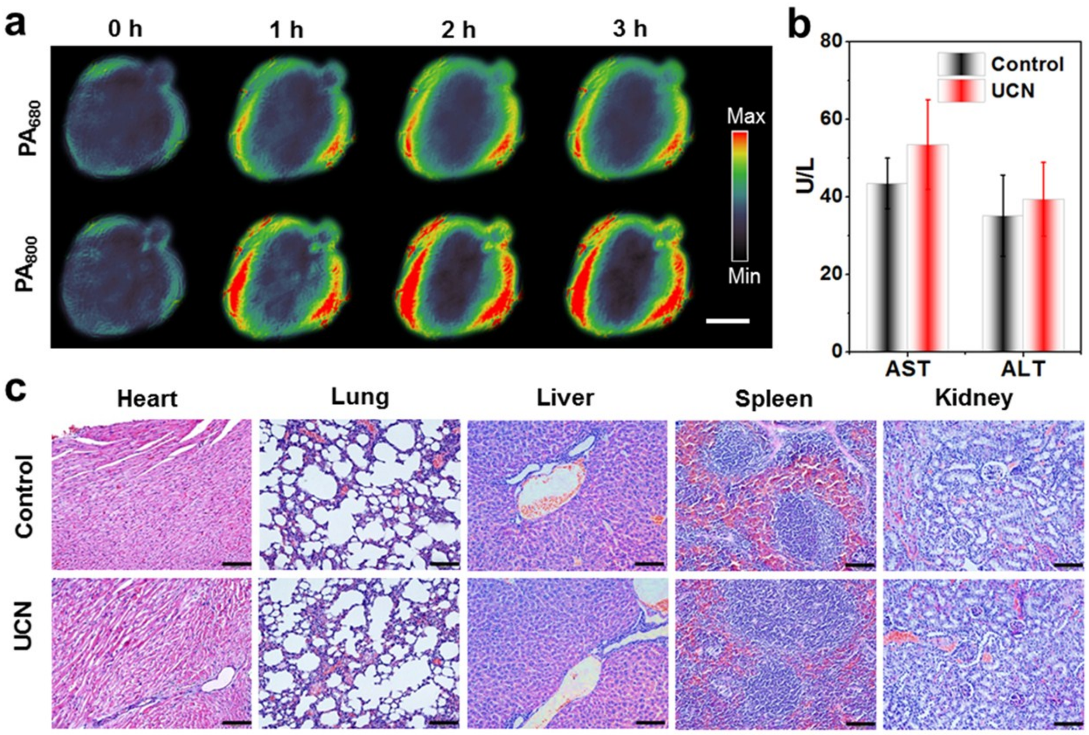

Figure S10. *In vivo* behaviors of UCN. (a) MSOT imaging of the mouse liver at 680 and 800 nm post UCN injection. Scale bar: 1 cm. (b) Serum ALT and AST activities after the administration of saline (control) and UCN (5 mg/mL, 100 µL). Values are expressed as mean ± SD (n = 5). (c) Histomorphological appearance of the major organs of mice (n = 3) after treatment with the saline only (control) or 24 h after administration of UCN. HE, scale bar: 100 μm.

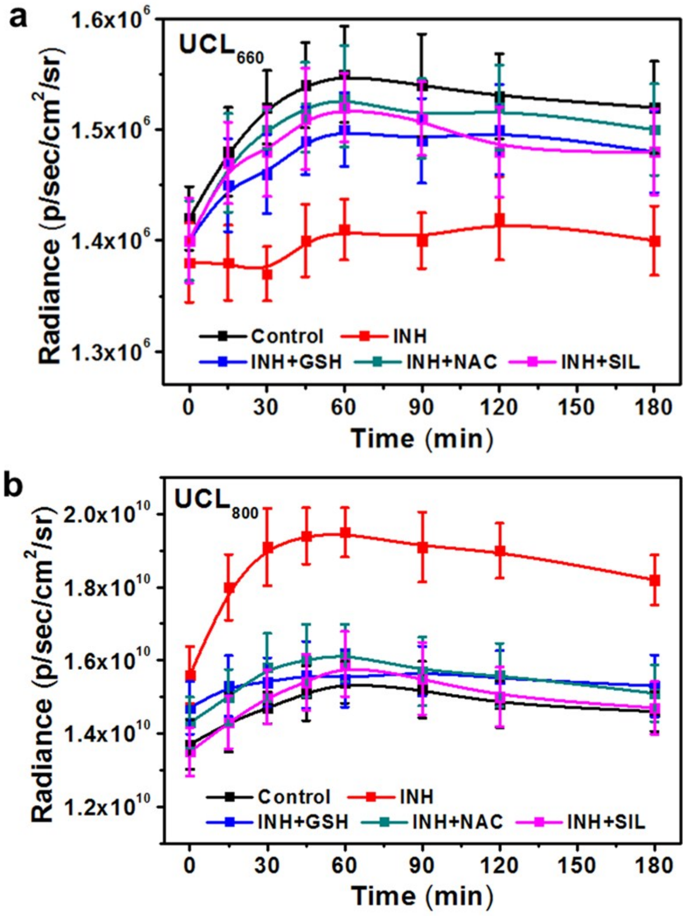

Figure S11. Dynamic upconversion luminescence signals of UCL_{660} (a) and UCL_{800} (b) at varied time points of UCN-pretreated (5 mg/mL, 100 μL) mice upon INH (100 mg/kg) stimulation with or without antioxidant therapeutic drugs (GSH, NAC and SIL, 200 mg/kg, respectively). Data represent means ± SD (n = 5). Ex. 980 nm.

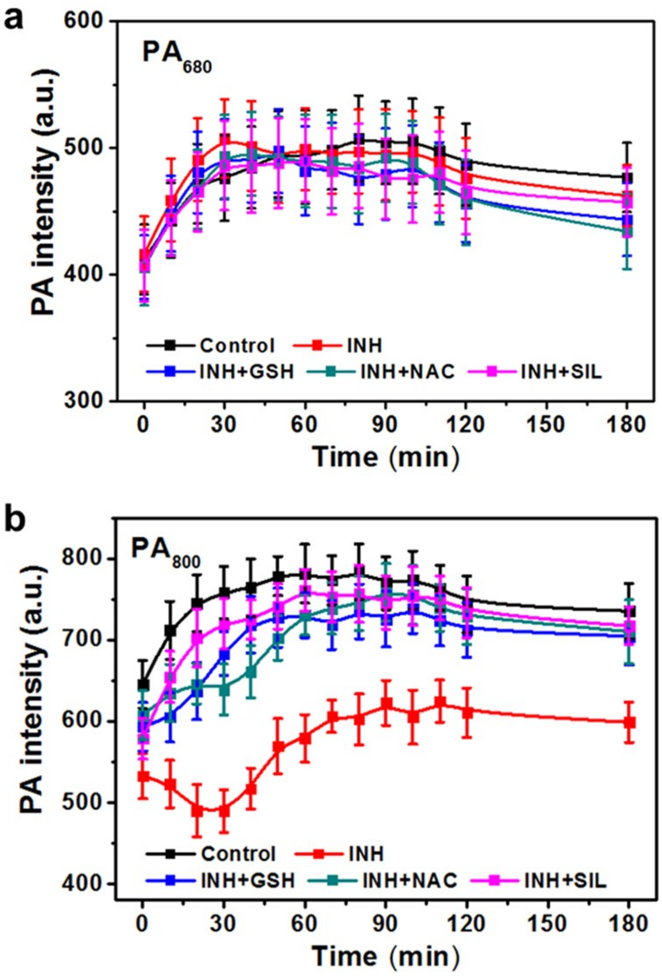

Figure S12. Dynamic photoacoustic signals of PA_{680} (a) and PA_{800} (b) at varied time points of UCN-pretreated (5 mg/mL, 100 μL) mice upon INH (100 mg/kg) stimulation with or without antioxidant therapeutic drug molecules (GSH, NAC and SIL, 200 mg/kg, respectively). Data represent means ± SD (n = 5).

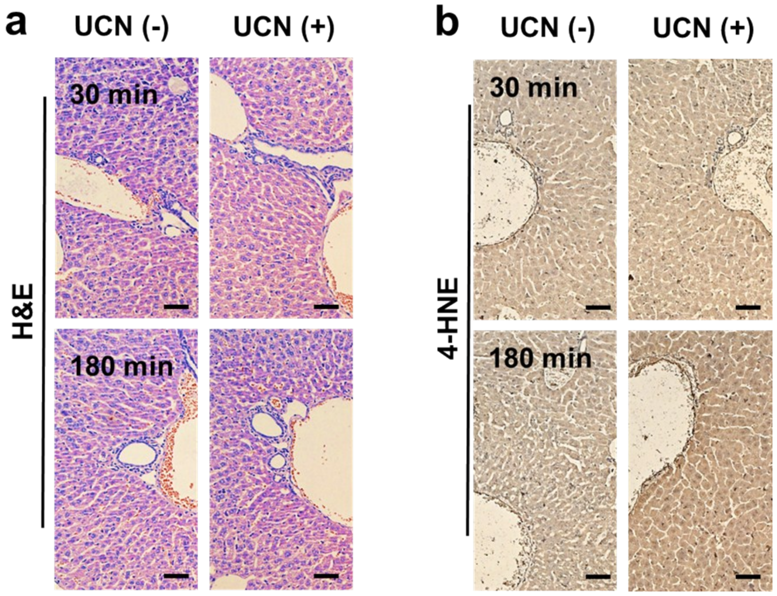

Figure S13. H&E staining (a) and immunohistochemical 4-hydroxynonenal (4-HNE) staining (b) of liver tissues at 30 and 180 min after INH treatment with or without antioxidant therapeutic drugs (n = 5). Scale bars: 100 μm.

EXPERIMENTAL METHODS

Chemicals and instruments

IR780, triphosgene, benzylamine, 4-(Hydroxymethyl)phenylboronic acid pinacol ester, pyridine, $Gd(CH_3CO_2)_3$, $Yb(CH_3CO_2)_3$, $Er(CH_3CO_2)_3$, $Tm(CH_3CO_2)_3$, oleic acid, 1-octadecene, NH_4F, NaOH, polyethylenimine (PEI, Mw 25000, branched) were purchased from Sigma-Aldrich and were used without further purification. Murine macrophages RAW264.7 and Kupffer cells were purchased from American-type culture collection (ATCC). Dulbecco's Modified Eagle Medium (DMEM), fetal bovine serum (FBS), penicillin-streptomycin and trypsin-EDTA were obtained from Invitrogen (Carlsbad, CA, USA).

^{1}H-NMR and were measured on a 500 MHz Bruker spectrometer. ESI-MS and high-resolution mass spectra were measured on a Waters Q-Tof Premier mass spectrometer (ESI measurements). High-resolution mass spectra were measured on a Waters Q-Tof Premier mass spectrometer (ESI measurements). High performance liquid chromatography (HPLC, Shimadzu) system was performed on an Alltima C-18 column of 250 × 10 mm at a flow rate of 3 mL min^{-1}. UV-Vis absorption spectra were measured using a Beckman coulter DU800 spectrometer. The upconversion emission spectra were recorded at an angle of 90° to the excitation laser (980 nm) and an optical SEC-2000 spectrometer coupled 2048 pixels CCD assay (ALS Co., Ltd, Japan). Transmission electron microscope (TEM) images were recorded using a FEI EM208S TEM (Philips) operated at 100 kV. Dynamic light scattering (DLS) measurement was performed by Brookhaven 90 Plus nanoparticle size analyzer.

The *in vitro* cytotoxicity was tested with a resazurin-based TOX8 assay kit and measured by a Bio-Tek EL-311 microplate reader. The upconversion luminescence (UCL) imaging in living cells

was recorded on a Nikon confocal fluorescence microscope (Nikon, Eclipes TE2000-E, Japan) equipped with 980 nm laser wide-field fluorescence add-on (EINST Technology Pte Ltd, Singapore). All the photoacoustic (PA) imaging experiments *in vitro* and *in vivo* were performed by using a real-time multispectral optoacoustic tomographic (MSOT) imaging system from iThera Medical GmbH (Neuherberg, Germany). The UCL imaging in living mice was performed with an IVIS Lumina II imaging system (Caliper Life Sciences, France) with 980 nm NIR laser irradiation. All animal experimental procedures were performed in accordance with the protocol approved by the Institutional Animal Care and Use Committee of Soochow University.

Synthesis of ROS probe (CyB). As shown in **Scheme S1**, the small-molecule H_2O_2 probe (CyB) was synthesized as follows according to the previously reported method with some slight modifications.[1]

*Synthesis of intermediate **2***. To flame dried two-neck round bottom flask containing triphosgene (254 mg, 0.85 mmol), a solution of (4-(4,4,5,5-tetramethyl-1,3,2-dioxaborolan-2-yl)phenyl) methanol (**4**) (100 mg, 0.43 mmol) dissolved in dry DCM (10 mL) was drop-wisely added. The solution was cooled to 0 °C using an ice-water bath, then pyridine (52 μl, 0.65 mmol) was added *via* syringe. Then the reaction was slowly warmed to room temperature, stirred for 3 h, followed by dilution with ethyl acetate (25 mL). The resulting mixture was partitioned with 1M HCl (2 mL) and the biphasic mixture was placed into a separatory funnel. The organic layer was washed with brine and water, dried over Na_2SO_4, filtered, and concentrated under reduced pressure. The crude product was used for the subsequent reaction without further purification. ^{1}H NMR ($CDCl_3$, 300 MHz) δ 7.83 (d, J = 8.1 Hz, 2H) 7.41 (d, J = 8.1 Hz, 2H), 5.32 (s, 2H), 1.36 (s, 12H).

Synthesis of intermediate CyA. IR780 (**3**) (134 mg, 0.2 mmol) was dissolved in dry acetonitrile,

then the solution was mixed with benzylamine (**4**) (44 μl, 0.4 mmol) and N, N–diisopropylethylamine (DIEA) (33 μl, 0.2 mmol). The reaction mixture was heated at 80 °C for 40 min under N_2 protection, then quenched with 0.1 M HCl. The mixture was evaporated and the residue was extracted with H_2O/DCM (3 × 10 mL), then combined the organic phase and dried over anhydrous Na_2SO_4. The solvent was removed to afford the crude product, which was then purified with silica gel column chromatography using DCM/0-5% MeOH as eluent to obtain the desired product CyA. Yield 62% (122 mg). ^{1}H NMR (500 MHz, DMSO-d6) δ 7.65 (d, J = 12.7 Hz, 2H), 7.45 (d, J = 7.0 Hz, 2H), 7.42 – 7.38 (m, 2H), 7.35 (d, J = 7.1 Hz, 1H), 7.30 – 7.21 (m, 4H), 7.06 (t, J = 7.4 Hz, 2H), 6.88 (d, J = 7.8 Hz, 2H), 5.67 (d, J = 12.9 Hz, 2H), 5.03 (s, 2H), 3.94 – 3.78 (m, 4H), 1.86 – 1.80 (m, 4H), 1.48 (s, 12H), 1.03 (t, J = 7.4 Hz, 6H). ESI-MS m/z: 610.5 $[M+H]^+$.

Synthesis of CyB. The intermediate **2** (118 mg, 0.4 mmol) and CyA (73 mg, 0.1 mmol) were dissolved in dry DCM (10 mL), then DIEA (66 μl, 0.4 mmol) was dropwise added in the mixture. The solution was stirred at room temperature for 24 h under N_2 atmosphere. The reaction mixture was washed with saturated NH_4Cl solution and brine, extracted with DCM then dried over anhydrous Na_2SO_4. The organic solvent was evaporated to obtain the crude product and then purified with HPLC using H_2O/50-100% in ACN as eluent to afford the product CyB. Yield ~10% (20 mg). ^{1}H NMR (500 MHz, $CDCl_3$) δ 7.65 (d, J = 12.7 Hz, 2H), 7.45 (d, J = 7.0 Hz, 2H), 7.42 – 7.38 (m, 2H), 7.35 (d, J = 7.1 Hz, 1H), 7.30 – 7.21 (m, 4H), 7.06 (t, J = 7.4 Hz, 2H), 6.88 (d, J = 7.8 Hz, 2H), 5.67 (d, J = 12.9 Hz, 2H), 5.03 (s, 2H), 3.94 – 3.78 (m, 4H), 1.86 – 1.80 (m, 4H), 1.48 (s, 12H), 1.03 (t, J = 7.4 Hz, 6H). ESI-MS m/z: 870.87 $[M+H]^+$; HR-MS m/z: 870.5394 (Calc. 870.5381) $[M+H]^+$, $C_{57}H_{69}BN_3O_4$.

Preparation of core-shell UCNPs. The $NaGdF_4$:Yb/Er/Tm (20/2/1%) core UCNPs were prepared by following a previously reported method.[2] Briefly, 2 mL of $RE(CH_3CO_2)_3$ (RE = Gd, Yb, Er and Tm) containing solution, 3 mL oleic acid and 7 mL 1-octadecene were added in a 50 mL three-neck flask. The ratio of Yb/Er/Tm is 20/2/1% and the total lanthanide amount is 0.4 mmol. The mixture was heated to 150 °C for 60 min before cooling down to room temperature. Subsequently, a methanol solution (6 mL) containing NH_4F (59.3 mg) and NaOH (40.0 mg) was added and stirred for 30 min at 50 °C. Then methanol was evaporated and the solution was stirred at 290 °C for 1.5 h under nitrogen atmosphere. The core nanoparticles were precipitated by ethanol after cooling down to room temperature, then collected through centrifugation after several times ethanol washing, re-dispersed in 5 mL hexane for subsequent shell coating.

To prepare $NaGdF_4$:Yb/Er/Tm@$NaGdF_4$ core-shell UCNPs, 3 mL oleic acid and 7 mL 1-octadecene were firstly added in a 50 mL three-neck flask, then 2 mL of 0.4 mmol $Gd(CH_3CO_2)_3$ methanol solution was added. The reaction was heated to 150 °C for 60 min before cooling down to room temperature. The as-synthesized $NaGdF_4$:Yb/Er/Tm core nanoparticles in 5 mL hexane were added along with a methanol solution (6 mL) containing NH_4F (59.3 mg) and NaOH (40.0 mg). The resulting mixture was stirred at 50 °C for 30 min. Then methanol was evaporated and the solution was kept at 290 °C for 2 h under nitrogen atmosphere. After cooling down to room temperature, the core-shell nanoparticles were precipitated using ethanol and collected through centrifugation several times after ethanol washing. Finally, the core-shell UCNPs were re-dispersed in 10 mL cyclohexane for the subsequent experiment usage.

UCNPs modification. The preparation of biocompatible PEI and PEG modified UCNPs for *in vivo* applications were carried out following **Scheme S2**.[3] Briefly, the as-prepared hydrophobic

oleate-capped UCNPs were precipitated using ethanol and re-dispersed in a 10 mL acid aqueous solution (pH = 3) adjusted by 0.1 M HCl. Then the solution was sonicated for 30 min following with vigorous stirring for 2 h. Then the aqueous solution was extracted with 30 mL diethyl ether to remove the oleic acid ligands for three times. Finally, the water dispersible ligand-free UCNPs were recuperated by centrifugation (18,000 rpm, 10 min) after precipitation with 20 mL acetone. The ligand-free UCNPs were re-dispersed in 10 mg/mL branched PEI (Mw. 25,000) solution by sonication, and adjusted to pH 6 with 6 M HCl. After stirring for 24 h at room temperature, the PEI-UCNPs were collected by centrifugation (18,000 rpm for 10 min), washed with water for several time, and re-dispersed in water. Then PEI-UCNPs were further functionalized with PEG-COOH by the coupling reaction.[4] Briefly, 100 mg of PEG_{3400}-COOH (Merck) were dissolved in water (5 mL) containing EDC (47.9 mg, 0.25 mmol) and NHS (57.5 mg, 0.5 mmol) to form the active succinimidyl ester for 30 min. Then the mixture was added slowly to 5 mL PEI-UCNPs (10 mg mL^{-1}) water solution with 30 min sonication.[5] After vigorous stirring overnight, the UCNPs@PEI-PEG was washed with DMF and water for several times respectively by centrifugation (18,000 rpm for 10 min) and re-dispersed in 5 mL PBS with the concentration of 10 mg mL^{-1}.

Preparation of UCNP@PEI-PEG/CyB nanoconjugates (UCNs). The ROS-response probe CyB was loaded on the polymeric surface of PEG-PEI-UCNPs by the hydrophobic interaction.[6] The DMSO solution of CyB (2 mg) was drop-wise added to the UCNPs@PEI-PEG water solution (10 mg) under sonication for 30 min. The mixture was then stirred vigorously overnight at room temperature. Finally, the UCNP@PEI-PEG/CyB nanoconjugates were collected by centrifugation (18,000 rpm, 10 min) followed by two times washing to remove excess reactant. The loading

amounts of CyB was determined by the UV-vis absorbance at 800 nm. Transmission electron microscope (TEM) and dynamic light scattering (DLS) measurements were performed to characterize the successful preparation of the nanoprobe, UCN.

Ratiometric detection of H_2O_2 *in vitro*.

The specificity of H_2O_2 sensing was firstly tested by incubating UCN (0.5 mg mL^{-1}) in 10% DMSO HEPES buffer with various reactive oxygen species (50 μM) that are commercial stock solutions available or prepared following previous methods[7, 8], including H_2O_2, $OONO^-$, ClO^-, TBHP, $BuO^•$, $^•OH$, NO and $O_2^{-•}$. After 30 min incubation at ambient temperature, the UV-vis absorption was determined for those solutions. The selectivity of various radicals were plotted by the ratio of Abs_{650}/Abs_{800}. For the upconversion luminescence (UCL) test of H_2O_2 response, the samples were added in a 1 cm cuvette and irradiated with 980 nm continues laser, then UCL spectra were recorded and the H_2O_2 response was further plotted by the ratio of UCL_{660}/UCL_{800}. The photoacoustic signals of UCN incubating with different concentrations of H_2O_2 were recorded by iThera MSOT imaging system from 680 nm to 980 nm.

The ratiometric PA plot (PA_{680}/PA_{800}) was also carried out to evaluate H_2O_2 response. The lifetime decay curves of free UCNPs and the nanoprobe before and after H_2O_2 reaction at 800 and 660 nm were determined on the lifetime spectrometer (FSP920). The optical energy transfer (OET) efficiency (E) from the donor (UCNP) to the acceptor (CyB) could be calculated based on the following formula: $E = 1 - UCL_{DA} / UCL_D$ (Where UCL_{DA} and UCL_D are the luminescence intensity of UCNPs in the presence and absence of loaded dye (*e.g.*, CyB), respectively).

Cell culture and cytotoxicity test. RAW264.7 and Kupffer cells were cultured in high-glucose DMEM containing 10% fetal bovine serum (FBS), penicillin (100 I. U. mL^{-1}) and streptomycin

(100 μg mL^{-1}) in a humidified atmosphere with 5% CO_2 at 37 °C. For the cytotoxicity assay, cells were seeded in 96-well plates (8,000 cells/well in 100 μL) and incubated for overnight. Different concentrations of UCN were added to the cell culture medium, respectively, then the cells were incubated for 24 h. The medium was removed and replaced with TOX8-contaning medium solution, incubated for another 3 h. The fluorescence at 590 nm was measured by a Tecan's Infinite M200 microplate reader with 560 nm excitation. Cell viability was expressed by the ratio of the fluorescence of cells incubated with UCN to that of control cells.

Cell imaging studies. For the cellular ROS sensing, RAW264.7 cells were cultured in the confocal dish and incubated with UCN (0.1 mg mL^{-1}), then lipopolysaccharide (LPS, 2 μg mL^{-1}) was treated to induce oxidative stress and H_2O_2 overgeneration. After 4 h co-incubation, both LPS-treated, NAC-treated and control groups in confocal dishes were washed, the cellular UCL imaging was performed in Nikon fluorescence microcopy upon 980 nm NIR light excitation (5 W cm^{-2}).

Moreover, MSOT imaging for these samples in different groups were also performed by encapsulation the cell lysates into PA phantom respectively, which contains two-channel polyurethane cylindrical, one for holding the control medium (DMEM) and the other for holding the cell sample with UCN treatment. The MSOT images and signals were finally recorded using a 128-element concave transducer array spanning a circular arc of 270° with the optimal excitation wavelength from 680 to 980 nm.

Animal model and dynamic biodistribution study of UCN. All animal experimental procedures were performed in accordance with the protocol approved by the Institutional Animal Care and Use Committee of Soochow University. The Balb/c nude mice (~ 6-8 weeks old) were purchased

from Shanghai Laboratories Animal Center in China. To study the biodistribution of UCN, the nude mice were intravenously injected with UCN (5 mg mL^{-1} in 100 μL saline) and were further sacrificed at different time points post administration. Major organs including heart, liver, spleen, lung, kidney, stomach and intestines were collected and washed with PBS (10 mM, pH = 7.4) for three times, and then UCL signals from UCN were recorded based on the IVIS Lumina II imaging system upon NIR light irradiation (E_x: 980 nm, E_m: 790/30 nm).

***In vivo* PA and UCL imaging**. To monitor the dynamic ROS production in the antituberculosis drug (INH)–induced acute hepatic disease model, mice were fasted overnight and intraperitoneal (*i.p.*) treated with sterilized saline solution with or without isoniazid (INH, 100 mg Kg^{-1}), respectively (n = 5). For hepatoprotective pharmacological assessments, INH-stimulated mice were pre-treated with the clinically used drugs including glutathione (GSH, 200 mg Kg^{-1}) inveavenously (*i.v.*) 5 min before INH treatment, with N-acetylcysteine (NAC, 200 mg Kg^{-1}) intraperitoneally (*i.p.*) 1 h before INH treatment, and Silymarin (SIL, 200 mg Kg^{-1}) intragastrically (*i.g.*) 12 h before INH treatment, respectively, thereby these hepatoprotective drugs could reach an effective plasma concentration.[9-11] Meanwhile, UCN (5 mg/mL in 100 μL saline) were injected intravenously before 1 h of INH stimulation to reach an effective liver accumulation, and the mice were then anesthetized with 3% isoflurane for UCL imaging at 660 nm and 800 nm on the IVIS Lumina II animal imaging system with specific filters (*e.g.,* 640/50 nm and 790/30 nm) upon 980 nm NIR light irradiation (10 W cm^{-2}). The real-time MSOT imaging was also performed from 680 nm to 980 nm after hepatoprotective drugs and UCN administration at different time points with 10 min interval. To dynamic and ratiometric profile the ROS status in the liver, both the time-resolved MSOT and UCL signals with pseudo-color processing were collected upon INH injection,

and the ROI intensities were recorded on the tomographic anatomical image of liver cross section in iThera software.

Histological and immunohistochemical analysis. The main organs including liver, heart, spleen, lung and kidney were resected and placed into 4% formalin solutions overnight at 4 °C, and all tissues were embedded in paraffin with 10 μm section. For the histological studies, these organ tissues were stained by hematoxylin and eosin (H&E). For the immunohistochemical staining, the samples were processed for 4-hydroxynonenal (4-HNE) staining based on the anti-4-HNE primary antibody (Abcam) at 1:500 dilution by following the manufacturer's methods. All images were acquired using an Olympus IX53 inverted fluorescence microscope equipped with a Nuance (CRi Inc.) hyperspectral camera capable of bright field full-color imaging.

Serum aminotransferases tests. The mice were anesthetized and the blood was collected from the vena cava at different time points (n = 5). Then heparinized serum was separated immediately to measure aspartate aminotransferase (AST) and alanine aminotransferase (ALT) activities by following the standard protocols. The liver function was evaluated by analyzing the relative level of these two indicators.

Data analysis. The signals of PA and UCL were measured by region of interest (ROI) analysis. Results were expressed as the mean ± SD unless otherwise stated. Significant differences can be determined using the Student's two-tailed t test where differences were considered significant (*p < 0.05, **p < 0.01, ***p < 0.001). All statistical calculations were performed using OriginPro 8.5 (OriginLab Corporation, USA).

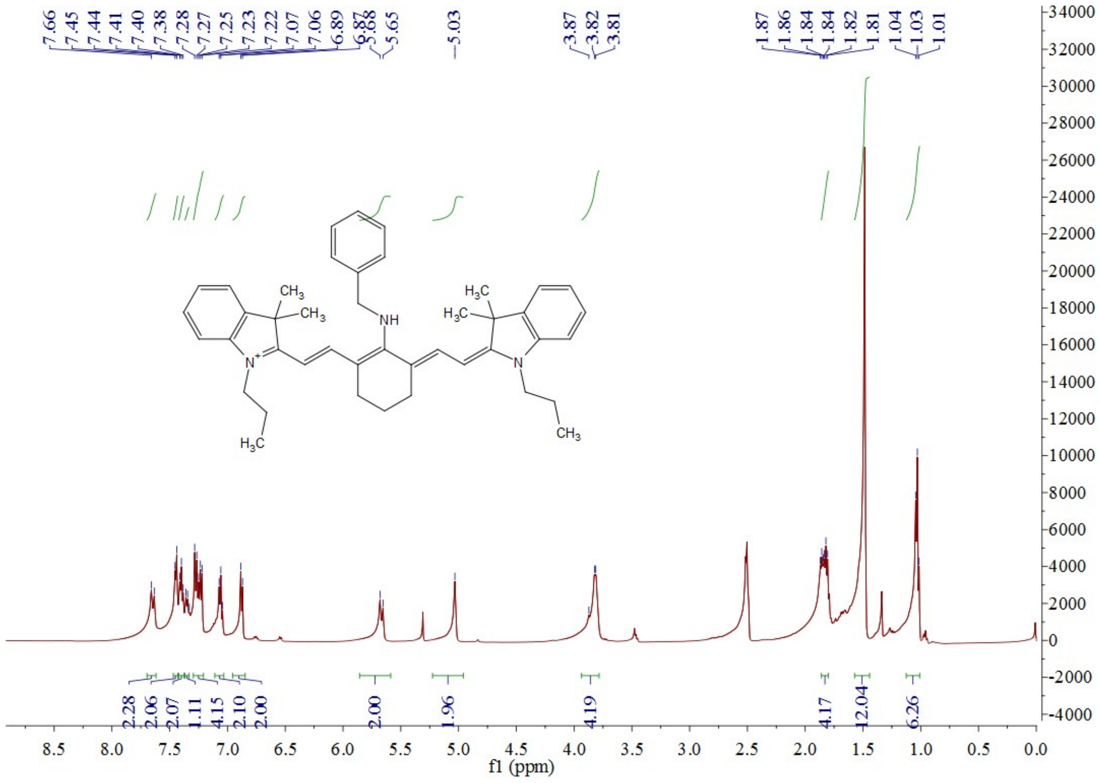

^{1}H NMR spectra of CyA (DMSO-d6)

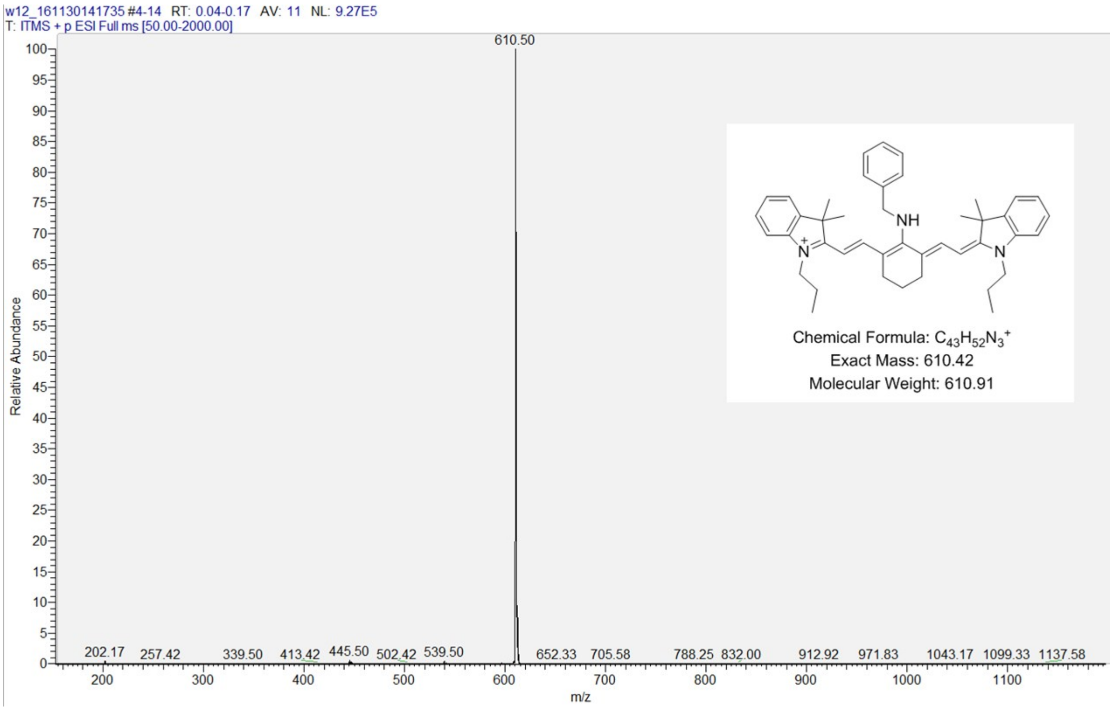

ESI-MS spectra of CyA

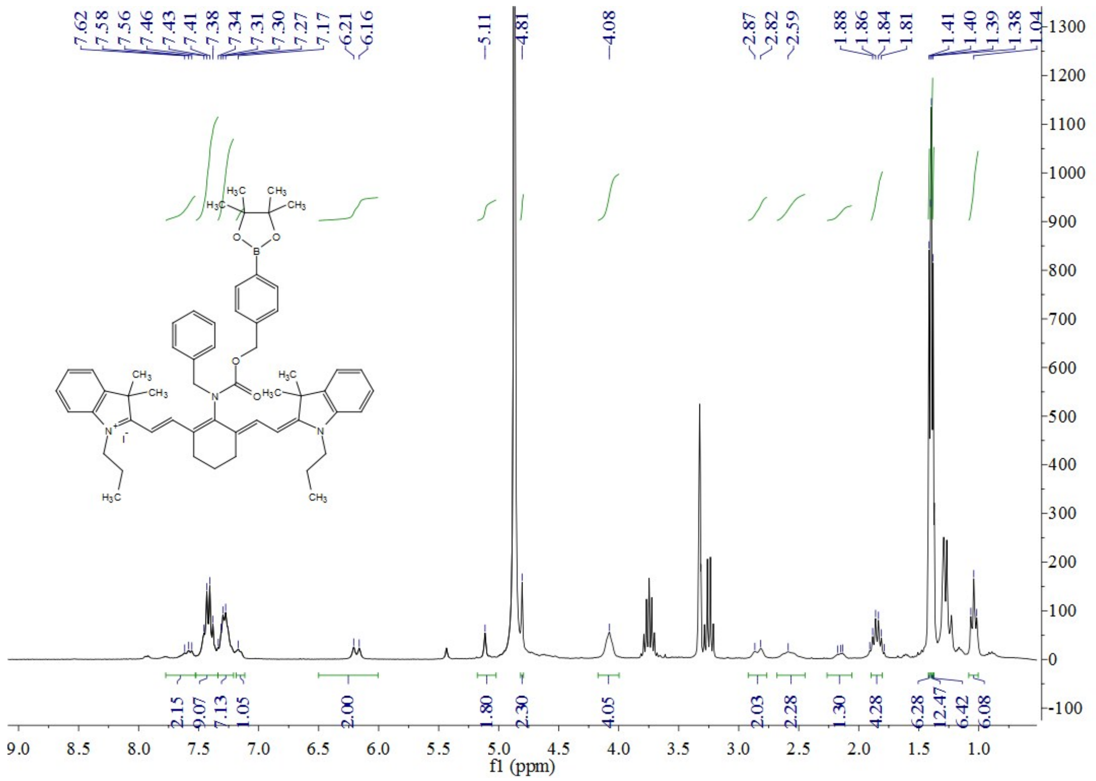

^{1}H NMR spectra of CyB (MeOD)

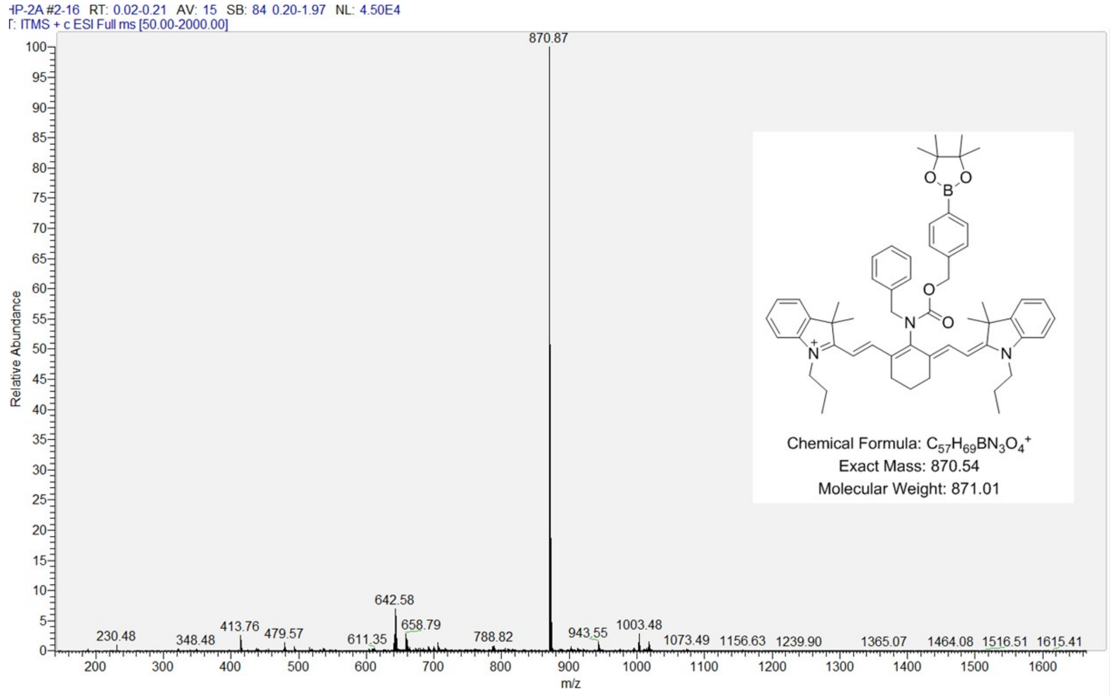

ESI-MS spectra of CyB

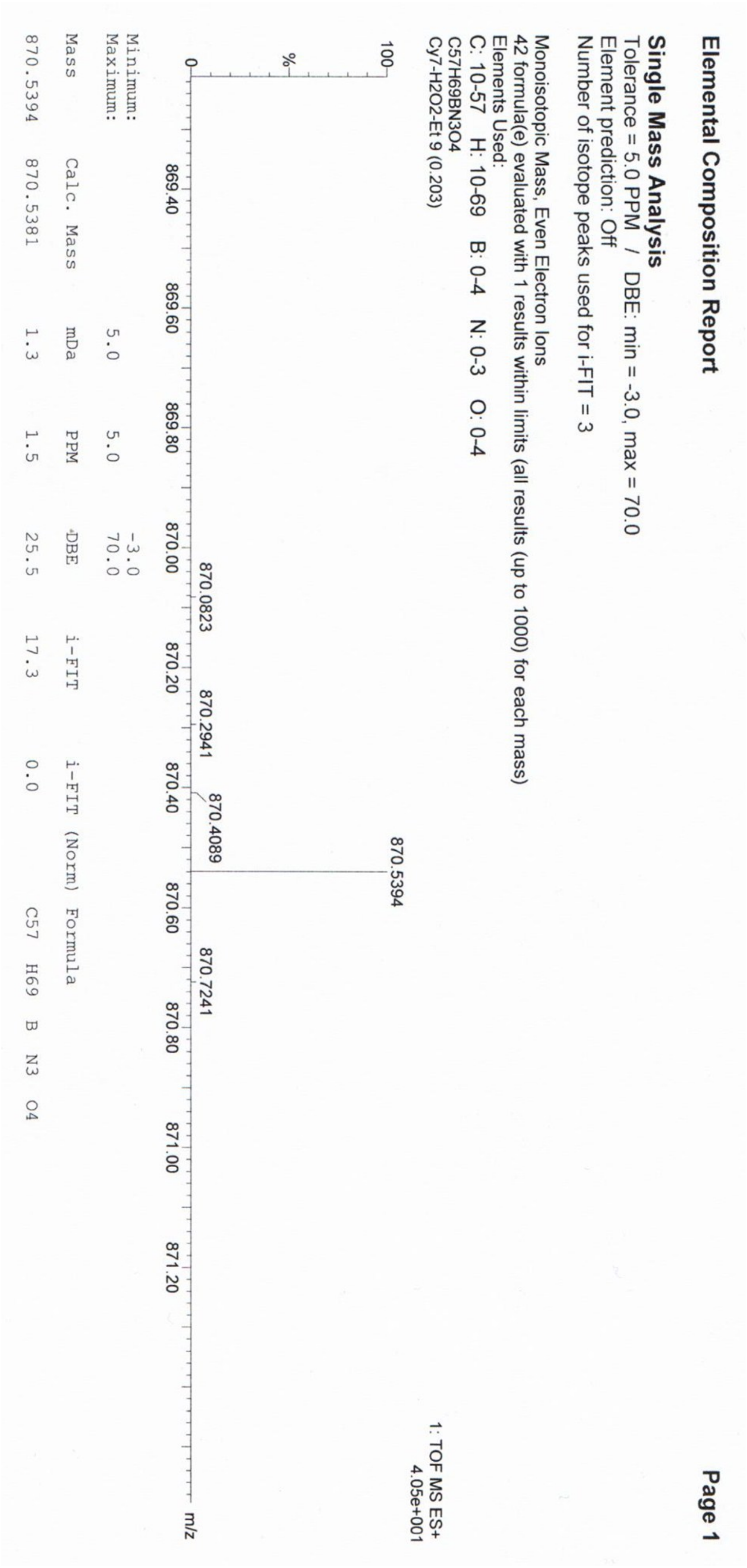

HR-MS spectra of CyB

References

1. D. Zhu, G. Li, L. Xue and H. Jiang, *Org. Biomol. Chem.*, 2013, **11**, 4577-4580.
2. F. Wang, R. Deng and X. Liu, *Nat. Protoc.*, 2014, **9**, 1634.
3. N. Bogdan, F. Vetrone, G. A. Ozin and J. A. Capobianco, *Nano Lett.*, 2011, **11**, 835-840.
4. A. Sedlmeier and H. H. Gorris, *Chem. Soc. Rev.*, 2015, **44**, 1526-1560.
5. X. Ai, C. J. H. Ho, J. Aw, A. B. E. Attia, J. Mu, Y. Wang, X. Wang, Y. Wang, X. Liu, H. Chen, M. Gao, X. Chen, E. K. L. Yeow, G. Liu, M. Olivo and B. Xing, *Nat. Commun.*, 2016, **7**, 10432.
6. Y. Liu, M. Chen, T. Cao, Y. Sun, C. Li, Q. Liu, T. Yang, L. Yao, W. Feng and F. Li, *J. Am. Chem. Soc.*, 2013, **135**, 9869-9876.
7. X. Jia, Q. Chen, Y. Yang, Y. Tang, R. Wang, Y. Xu, W. Zhu and X. Qian, *J. Am. Chem. Soc.*, 2016, **138**, 10778-10781.
8. K.-i. Setsukinai, Y. Urano, K. Kakinuma, H. J. Majima and T. Nagano, *J. Biol. Chem.*, 2003, **278**, 3170-3175.
9. S. Attri, S. Rana, K. Vaiphei, C. Sodhi, R. Katyal, R. Goel, C. Nain and K. Singh, *Hum. Exp. Toxicol.*, 2000, **19**, 517-522.
10. C. Saito, C. Zwingmann and H. Jaeschke, *Hepatology*, 2010, **51**, 246-254.
11. S. Eminzade, F. Uras and F. V. Izzettin, *Nutr. Metab. (Lond.)*, 2008, **5**, 18.
